# Liposomal Drug Delivery Systems for Cancer Therapy: The Rotterdam Experience

**DOI:** 10.3390/pharmaceutics14102165

**Published:** 2022-10-11

**Authors:** Mohamadreza Amin, Ann L. B. Seynhaeve, Majid Sharifi, Mojtaba Falahati, Timo L. M. ten Hagen

**Affiliations:** 1 Laboratory Experimental Oncology, Precision Medicine in Oncology (PrMiO), Department of Pathology, Erasmus University Medical Center, 3015 GD Rotterdam, The Netherlands; 2 Nanomedicine Innovation Center Erasmus (NICE), Erasmus University Medical Center, 3015 GD Rotterdam, The Netherlands; 3 Student Research Committee, School of Medicine, Shahroud University of Medical Sciences, Shahroud 3614773955, Iran; 4 Department of Tissue Engineering, School of Medicine, Shahroud University of Medical Sciences, Shahroud 3614773955, Iran

**Keywords:** liposomes, hyperthermia, active targeting, vascular targeting, vascular permeabilization, intravital microscopy, temperature sensitive liposomes, cancer therapy, drug delivery, smart drug delivery system, The Netherlands

## Abstract

At the Nanomedicine Innovation Center (NICE) at the Erasmus MC in Rotterdam, we have approached the treatment of cancer by starting with a vision of first establishing a platform that enables us to overcome the low levels of drugs delivered to tumors and the issue of dose-limiting toxicity. Showing that a reduction of the volume of distribution, and a lowering of toxicity and side-effects, accompanied by augmented intratumoral drug delivery, could change outcomes in patients, paved the way to target, not only localized disease, but also systemic and metastasized cancers. In particular, the detailed studies with intravital microscopy we performed at NICE provided us with the necessary insights and affected to a large extent our program on liposome-based cancer therapy. Together with our experience with the loco-regional treatment of cancer, this helped us to develop a program that focused on the subsequent aspects discussed here. We recognized that passive accumulation of nanoparticles was not as effective as previously believed and undertook to improve the local accumulation by changing the tumor pathophysiology and, in particular, the vascular permeability. We added the targeting of liposomes using vascular and tumor directed moieties, to improve cellular drug delivery. To improve payload delivery, we studied the modification of liposomes with phospholipids that help passive drug release and augment cellular accumulation. Second, and importantly, modification of liposomes was undertaken, to enable triggered drug release. The capability for modifying liposomes to respond to a trigger, and the ability to now apply an external trigger (e.g., hyperthermia) and specifically reach the tumor volume, resulted in the current smart drug delivery systems. Our experience at NICE, after a few decades of research on lipid-based nanoparticles, shows that, after the first liposomal formulation registered for clinical application in cancer therapy, further developments quickly followed, while further clinical applications lagged behind. Now we need to focus on and make the next steps towards the clinic, to fulfil the promise that is found there.

## 1. Introduction

According to a global report, the population of cancer patients in the world is expected to increase to 22 million by 2035 [1]. Despite the development of surgical procedures and combined radiation-based therapies, clinical application and treatment results indicate that chemotherapy is increasingly used as the common approach for cancer treatment. However, the success of chemotherapy depends on several factors, such as the dose, toxicity, personalized care, optimal drug regimens, access to the tumor site, and drug targeting [2,3]. An important aspect of chemotherapeutics is the unfavorable efficacy to toxicity ratio. Chemotherapy is typically associated with severe side effects, which not only have unfortunate consequences for the standard of living of patients, but also preclude higher dosing, to compensate for the relatively poor accumulation of chemotherapeutics at the target site, i.e., the tumor.

Formulating chemotherapeutics in nanoparticles is considered a promising approach that, not only makes administration of poorly water soluble compound possible [4], but also improves drug delivery to tumors and reduces the adverse side effects associated with cytotoxic chemotherapeutics [5,6,7]. Improved pharmacokinetics, and consequently reduced side effects [4,6,7,8,9,10], stabilizing the payload [11,12,13], and the possibility of the co-delivery of different compounds in one nanoparticle are the key benefits of nano-drugs, particularly liposomes, that have emerged in the clinic. However, it is now generally accepted that the clinical advantage of nanomedicine is the more manageable post-treatment side effects and not necessarily an improved anti-tumor response [14,15].

Since Maeda and his co-workers reported that nano-sized particles could preferentially extravasate into tumor interstitium because of the leaky tumor vasculature and remain there, due to the lack of a functional lymphatic drainage system [16], this phenomenon, also known as enhanced permeation and retention effect (EPR), has become the cornerstone of the design and application of nanomedicines to treat cancer. Since 1960, more than 59,000 original research papers on the use of nanocarriers have been published, of which more than 1600 are traceable in the Netherlands.

Unfortunately, the heterogeneous nature of tumors, not only between different tumors [17], but more importantly within an individual tumor [18,19], makes EPR based drug delivery less reliable. Strikingly, a considerable part of the research done today on nanoparticles for drug delivery relies on the EPR as a means to reach individual tumor cells. Other factors related to the tumor microenvironment formulation of nanoparticles have also added to the controversy surrounding the use of NPs for drug delivery; while a stable composition and stealthiness is desired, to promote the pharmacokinetics of chemotherapeutics in long circulating nanoparticles, these two characteristics hamper cellular drug delivery, due to a limited drug release and/or poor interaction and uptake of nanoparticles by the targeted cells. These observations prompted the nanomedicine community to explore other alternative approaches to improving cellular drug delivery or to bypassing the EPR, for a more effective targeting of tumors [20,21,22].

**Figure 1:**
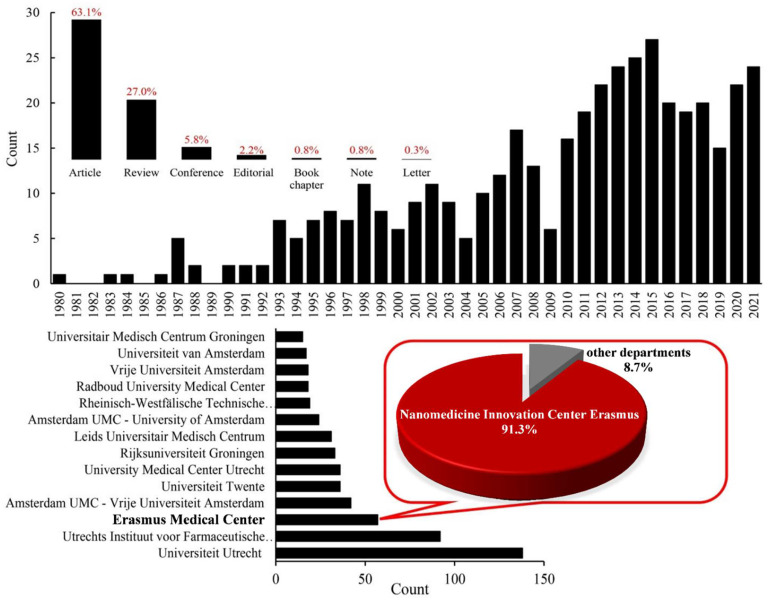
Chronological increase in the scientific attention on liposomes for cancer therapy in the Netherlands, Erasmus Medical Center, and the Nanomedicine Innovation Center Erasmus (NICE), based on reports from Scopus, PubMed, and Web of Science sites.

At the Erasmus Medical Center, the Nanomedicine Innovation Center Erasmus (NICE) is particularly involved in research towards improving drug delivery to tumors; either through manipulation of the tumor microenvironment, through surgical approaches, or by using smart drug delivery systems, mostly focused on liposomes (Figure 1). An important surgical-based development was the application of so-called isolated limb perfusion (ILP), where only the part of the body that contains the tumor is exposed to the chemotherapeutic. This is achieved by surgical separation of the limb with the to be treated tumor from the rest of the body for the duration of the treatment.

Since the introduction of liposomes as a drug delivery system by Gregory Gregoriadis in the early 1970s, liposomes have become one of the most well studied drug delivery systems [23]. Their ease of preparation and modification, biocompatibility, biodegradability, low immunogenicity, and simultaneous transport of both hydrophilic and lipophilic compounds make liposomes a versatile drug delivery system, with several commercialized products, such as liposomal doxorubicin (Doxil^®^, Janssen, PA, USA), liposomal vincristine (Marqibo^®^, Acrotech Biopharma, NJ, USA), Liposomal irinotecan (Onivyde^®^, Ipsen Biopharmaceuticals, Paris, France), and liposomal daunorubicin plus cytarabine (Vyxeos ^®^, Jazz Pharmaceuticals, Athlone, Ireland). Since 1980, there has been a growing number of scientific studies performed in the Netherlands using liposomes to treat various cancers (Figure 1). The Nanomedicine innovation Center Erasmus (NICE) has also been involved in the development of different liposome-based drug delivery systems to treat cancer (Table 1).

**Figure 2:**
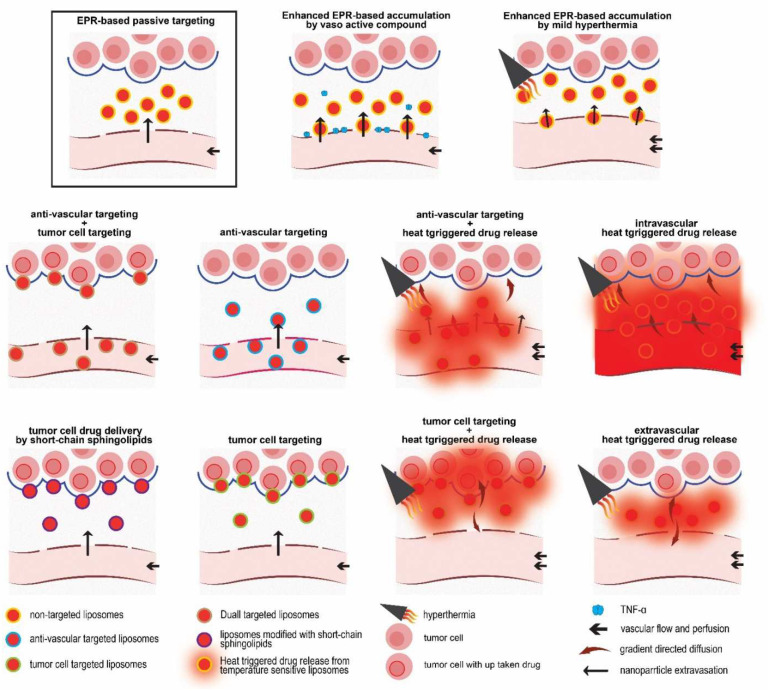
Multifaceted strategies employed at the Nanomedicine Innovation Center Erasmus to improve the efficiency of drug delivery to malignant cells.

Despite the favorable responses in vitro and in vivo, multidisciplinary approaches to treating cancer with a more uniform drug distribution appear challenging. Here at NICE, we have implemented multifaceted strategies (Figure 2) and have taken effective steps in this direction, which will be discussed further below. In this review, we place our endeavors in the context of other developments with respect to nano-sized liposomal drug delivery systems for the treatment of cancer.

## 2. Visualizing the Intratumoral Fate of Nanomedicines by Intravital Microscopy

**Figure 3:**
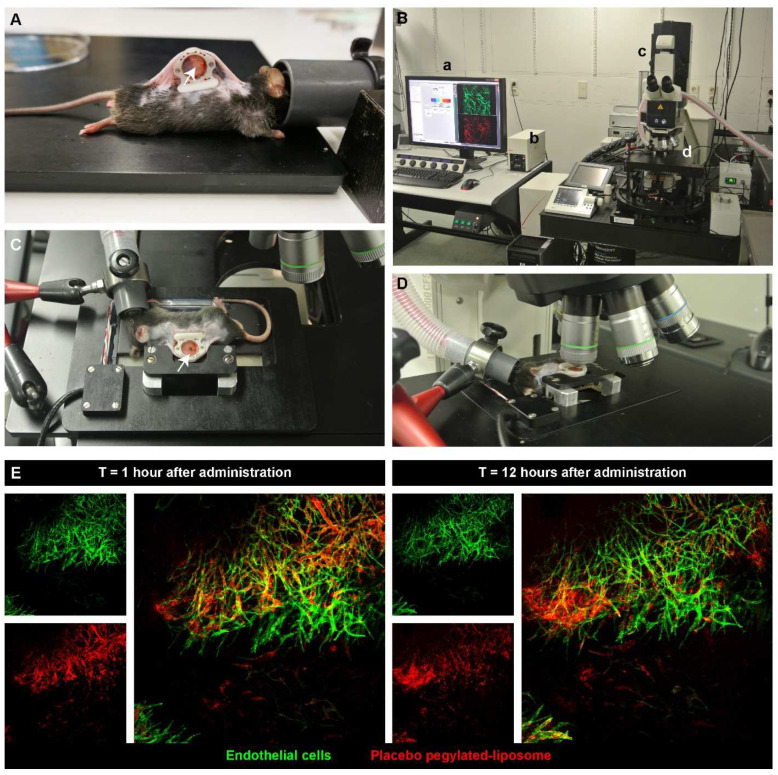
Imaging of a tumor by intravital microscopy. (**A**), Mouse after transplantation of a Lewis lung carcinoma (arrow) in a dorsal skinfold chamber. (**B**), Intravital microscope set up, a = computer and software, b = fluorescent light for fast scanning, c = confocal/multiphoton microscope, d = adapted table with heating platform and anesthesia connection. (**C**), Mouse with a B16BL6 melanoma tumor (arrow) fixated on a heated platform, (**D**), Mouse under the microscope ready for imaging, (**E**), intravital images of a Lewis lung carcinoma in a transgenic mouse with green fluorescent endothelial cells and injected with a red fluorescent placebo pegylated liposome. Images are taken 1 and 12 h after systemic administration.

Pharmacokinetics and dynamics, by measuring drug or lipid contents, are in general assessed in the blood and the entire tumor and organs of interest. However, measuring the overall drug and/or nanocarriers content does not necessarily reflect the intratumoral behavior or correlate with tumor response. In order to become active, an encapsulated payload needs to dissociate from the carrier, to become bio-available and then reach and interact with its target. For instance, doxorubicin (DXR) needs to enter the cell nucleus and intercalate with DNA to induce its main effect. In vitro evaluation of carriers, and the uptake and release of drugs, can be performed using live cell imaging, in which the process is carried out in living cells; as a detailed time-lapse can be made, without fixation artifacts [44]. To explore the fate of nanoparticles and their contents, in vivo intravital imaging is used at the Erasmus MC. Intravital imaging of a tumor implanted in an animal is an excellent tool for observing the real-time and longitudinal tumoral fate of the carrier, released drug, or other biological phenomenon taking place in a tumor, in a 4D (XYZ, spatial dimensions, and T, time dimension) manner. This technology has distinct advantages, as it allows high-resolution and continuous imaging over hours and even days, without further interference. Drugs, nanocarriers, tumor cells, and even normal cells, by using transgenic animals, can be given a fluorescent marker, creating an entire field of possibilities for in-detail imaging. At NICE, we employ an adapted dorsal skinfold chamber, with a tumor implanted in transgenic mice that have fluorescent labels in the cells of the vasculature [45,46,47]. Briefly, the skin flap of the animal is sandwiched and immobilized between two frames, using screws and sutures (Figure 3). In the front window, a transplantable area is created, by removing the skin and muscle layer and exposing the fascia, in which a tumor cube of 1 mm^3^ is implanted. The frames are closed off from the environment with a cover glass, and the tumor and tumor-associated vasculature develops and grows in this area. Blood flow is made visible using a bright field; however, small molecules such as Hoechst or larger particles such as fluorescent-labeled dextrans can also be injected, to allow a distinction between functional blood conducting vessels and still developing or necrotic vessels, and the permeability of the tumor-associated vasculature [46,48,49] (Figure 3). Cytotoxic agents can be fluorescently-labeled, and various known chemotherapeutics, such as doxorubicin, idarubicin, and mitoxantrone, and the model drug carboxyfluorescein, are intrinsically fluorophores [24,50,51,52]. Moreover, using different fluorescent lipophilic markers, such as rhodamine-PE, topfluor-PE, NBD-PE, DiD, or DiO, the nanoparticle itself can be made fluorescent [35,53,54]. Importantly, as can be seen in this review, intravital imaging allows for the imaging of the tumor and surrounding tissues as a whole; and because of the optimization of the window and microscope setup, processes in the living mouse can be studied at a subcellular level. Together, this gives insights into the pharmacokinetics, intratumoral kinetics, interactions with cells, and intracellular processing and trafficking of the nanosystem and drug.

Using intravital microscopy, we can image the entire tumor or focus on specific regions and even observe details in the cellular cytoplasm and nucleus. In this way, it was observed in vivo that Doxil was taken up intact by the cell, as the green lipophilic marker DiO overlapped with the red fluorescence of doxorubicin in the cytoplasm of tumor cells [50]. These results indicated that the stable formulation of a nanoparticle may indeed benefit the circulation time but also hinders the bioavailability of the contents. Moreover, despite the endothelial gap formation [55,56] and vascular permeability [48] in the tumor-associated endothelial lining, the extravasation of conventional liposomes of around 100 nm was minimal at best and very heterogeneous throughout the tumor. Larger liposomes at 400 and 800 nm predominantly remained inside the tumor-associated vasculature [50]. As will be discussed later, liposomes could be decorated with different ligands that specifically target the developing vasculature and/or cells inside the tumor. Intravital microscopy enabled us to monitor endothelial binding or an increased intracellular drug uptake compared to the non-targeted formulation [35,52,57]. Additionally, the possibility of augmented nanoparticle accumulation, through rendering the endothelial lining more permeable (i.e., more leaky) or through a combination of nanoparticle systems that can release their contents on demand with an applicable trigger, has been pursued, and the findings are discussed below.

In conclusion, the use of intravital microscopy allows us to investigate the tumoral fate of the drug, as well as the carrier itself, as it is possible to monitor the release rate, uptake, retention, intracellular location, and possible efflux in a timely manner and correlate these with tumor response, or the lack thereof. More importantly, this animal setting has provided invaluable insights for improving drug delivery and testing new systems. For instances, to apply heat locally in the dorsal skinfold chamber, an external circular electric heating coil was constructed, to fit into our chamber holder [58]. Several heating schedules (i.e., before, during, or before and after injection of liposomes) have been investigated, and via intravital microscopy, the drug release and uptake kinetics was evaluated. Using intravital microscopy, we could visualize and compare, as will be discussed later, the drug delivery using different thermosensitive liposomes (TSL).

## 3. Augmentation of Vascular Permeability by Tumor Necrosis Factor α

The heterogeneous and largely absent EPR effect in human tumors, along with characteristics of the tumor pathophysiology that work against nanoparticle accumulation (such as excesses of the extracellular matrix, high interstitial fluid pressure, solid stress) [59,60], prevent the homogeneous delivery, distribution, and deep penetration of large molecules and, most importantly, nanosized carriers. Therefore, a variety of strategies have been designed to overcome these hurdles.

Limiting the distribution volume from the whole body to a leg or an arm, as is accomplished in isolated limb perfusion, could significantly improve tumor response and avoidance of amputation in patients with advanced melanoma or sarcoma, when melphalan was administered in combination with TNF-α, compared to treatment with melphalan alone [61,62,63]. Investigating the potential of TNF in isolated limb perfusion and the mechanisms behind this increased antitumor effect, we observed that the addition of TNF, as well as other vaso-active agents such as histamine or interleukin-2, caused local edema and hemorrhage, and by doing so caused more drug to accumulate in the tumor [64,65,66,67,68]. Angiograms taken before and after TNF-based ILP showed that tumor-associated vessels were specifically affected, while normal vessels remained intact [69].

Clearly, nanosystems that can limit the volume of distribution, in combination with agents or other stimuli, to improve vascular leakage, can be used to improve treatment outcomes, since an important limitation of liposome-based cancer therapy is its poor intratumoral distribution [70]. The transport characteristics of nanosized liposomes—ones around 100 nm are mostly used—along with tumor status are the main reasons for this limitation [71]. We reported that administration of low-dose TNF-α, in combination with PEGylated liposomal doxorubicin (PLD), provided a significantly higher DXR delivery in soft tissue sarcoma in BN rats, resulting in a stable tumor volume compared to treatment without TNF-α [72,73]. Histologically, the levels of apoptotic and necrotic cells were so high that, from a pathological view, a partial and complete response was established in the combination treated animals. We demonstrated that the use of TNF-α in the first dose had no effect on the concentration of liposomal DXR in the tumor, while in subsequent doses, the amount of DXR delivered to the tumor was increased up to three times [73]. Addition of low dose TNF specifically stimulated the permeability of the tumor-associated vasculature for liposomes, whereas no increased DXR distribution was observed in the vital organs [73]. Similarly, Brouckaert, et al. [74] were able to show degradation of the endothelial lining by TNF-α with simultaneous use of TNF-α and PLD, which increased the drug accumulation in the tumor compared to the TNF-α-free group. This increased accumulation was associated with a reduced growth rate of B16BL6 melanoma tumors in C57BL/6 mice receiving PLD in combination with TNF-α. In this study, the distribution of DXR in vital organs, except in the spleen, showed that the presence of TNF-α had no impact on the accumulation of drug in the vital organs [74]. We also treated B16BL6 melanoma bearing animals with PLD, alone or in combination with TNF-α, and found a significantly higher drug accumulation and tumor response in animals treated with the combination [50]. However, whereas the effect of TNF in BN soft tissue sarcoma was realized after three administrations, the increased accumulation in the B16BL6 melanoma was already observed within 12 h of the first treatment. This indicates the important role of the tumor-associated vascular make-up, as the B16BL6 tumors were better vascularized compared to BN soft tissue sarcomas. However, to accomplish an improved TNF-induced intratumoral liposomal distribution, the size of the liposomes plays an important role. In the same report, we showed that co-administration TNF with 100-nm liposomes resulted in a higher drug accumulation to a murine melanoma tumor, compared to 100-nm liposomes alone (6.3-fold 12 h after administration, and 5.5-fold at 24 h). Co-administration of mTNF and 400 nm liposomes resulted in a 5.1-fold enhanced accumulation at 12 h and 9.2-fold at 24 h, compared with liposomes alone. More to the point, the drug delivery when using 100-nm liposomes, even without combination with TNF, was significantly higher compared to 400-nm liposomes. Moreover, TNF contributed to a more homogenous drug distribution, so that a larger tumor area took up a greater therapeutic concentration of DXR.

## 4. Application of Heat- and Temperature-Sensitive Liposomes

As stated earlier, the anti-tumor efficacy of chemotherapeutics carried by nanoparticles is hindered by the heterogeneous nature of tumors and the poor penetration of nano-sized vehicles into the depth of the tumor; both impair the preferential accumulation and homogeneous distribution of nanoparticles inside tumors [33,75]. In addition to this, an encapsulated drug is not bioavailable to cells and needs to dissociate to become active. These factors necessitate alternative approaches to enhancing the intratumoral drug delivery of bioavailable chemotherapeutics. One approach to enhancing the preferential drug release inside a tumor is the use of stimuli-sensitive carriers that, in response to an internally (reduced pH [76,77,78], elevated enzymatic [79], or redox activity [80]) or externally applied stimuli (magnetic and electric fields [81,82], ultrasound [83,84,85], light [86,87], or heat [51,88,89]), release the payload. While drug delivery based on endogenous stimuli may enhance the drug release, these methods do not necessarily improve drug delivery to the tumor region and mostly rely on passive accumulation through the EPR effect. Whereas, application of an exogenous stimulus enables a spatiotemporally controlled drug release. Furthermore, stimuli such as heat or ultrasound can increase the vascular permeability and improve the nanoparticle extravasation and accumulation inside tumors [90,91].

Mild hyperthermia is an advanced and conveniently applicable stimulus that, not only can be used for triggered drug release, but also has several therapeutic functions, including the following:

(a) Synergistic increase in the sensitivity of malignant cells to radiotherapy and chemotherapy [92,93].
(b) Increasing cell death in the hypoxic area in deeper, i.e., less oxygenated, regions of the tumor [94,95,96].
(c) Impairing the cellular mechanisms that repair DNA damages [97].
(d) Stimulation of the immune system towards an anti-tumor response [98,99,100,101].

These therapeutic potentials have made hyperthermia a treatment modality or a neoadjuvant therapy against cancer [102,103,104]. For more details we refer readers to Issels et al. [105].

Additionally, mild hyperthermia (around 42 °C) has proven advantages for manipulating the tumor microenvironment in favor of drug delivery to the tumor, by increasing the perfusion of the heated area and enlarging the gaps between vascular endothelial cells, which improves the vascular permeability [88,106,107,108]. We have previously demonstrated a reversible enlargement in gaps between endothelial cells, which remained for up to 8 h, upon application of mild hyperthermia to different tumor models [88]. Heat also increases the cellular permeability and drug uptake by the cells [97], while it physically increases the diffusion rate.

Given these biological impacts, the combination of mild hyperthermia with heat-triggered drug release is a convenient method that, not only provides on-demand drug release, but also enhances intratumoral and intracellular drug delivery and can enhance anti-tumor responses. For a more comprehensive review, we encourage readers to read our recent review on smart drug delivery systems [59,109,110].

**Figure 4:**
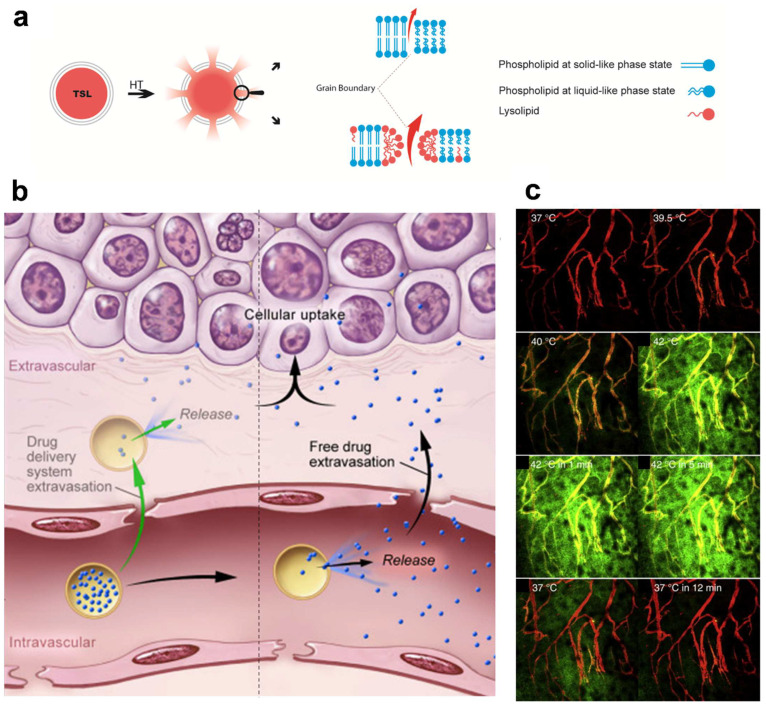
Schematic representation of the content release from temperature sensitive liposomes (TSL). (**a**) Represents the mechanism of drug release from different TSL formulations, upon formation of the grain boundaries between phospholipids domains coexisting in gel-like and liquid-like states at the transition temperature of the phospholipid bilayer (reprinted from [109] under the Creative Commons license. (**b**) Represents the application of TSL in extravascular or intravascular drug release settings (reprinted from [113] under the Creative Commons license). Panel (**c**) illustrates the in vivo release behavior of Rho-PE labeled TSL (red) encapsulating a self-quenched concentration of carboxyfluoprescein (CF, 100 mM), before, during, and after application of heat. While, before heat, TSL does not release significantly observable CF, heat triggers the CF release, which is followed by diffusion into the tumor interstitium. After heating, since CF has no specific affinity to tumor cells, it diffuses back into the blood and washes away (reprinted with permission from [24]).

Among the different temperature sensitive nanoparticles, the use of liposomes is more advanced and has proceeded to the clinical stage of development. The intrinsic characteristics of phospholipids, in exhibiting a thermotropic phase transition behavior from gel-like to liquid-like crystalline states, and which is accompanied by changes in the structure and permeability of the lipid bilayer at the transition temperature (Tc), make TSL a unique drug delivery system for heat triggered drug delivery; while it provides plenty of capacity to shuttle chemotherapeutics to the target site. It has to be taken into account that the mechanism of drug release from TSL is not, in general, related to the increased permeability of membranes in the liquid-like crystalline state, but is attributed to the formation of “grain boundaries” between the domains of phospholipids exhibiting gel-like phase (with a bilayer thickness of 5.0–5.5 nm) and the planes of phospholipids representing liquid-like phase (with a bilayer thickness of 4.0–4.5 nm), with both coexisting in a liposome membrane at the transition temperature [111,112] (Figure 4a).

Since the pioneering studies of Yatvin and coworkers [114], using DPPC (Tc: 41 °C)-based liposomes for heat triggered drug delivery upon application of mild hyperthermia, DPPC-based liposomes have subjected to a variety of modifications, such as the addition of DSPC to enhance heat sensitivity [27,115,116,117], the addition of ganglioside GM1 (GM1) [118] or polyethylene glycol (PEG) [119] to increase longevity, or cholesterol to decrease premature release during circulation [26,119,120]. In line with optimizing TSL, we found that, as in non-TSL, incorporation of 5 mol% mPEG2000-DSPE was an optimum content of PEG-conjugate [24,119], which not only promotes the PK of TSL but also assists with the release from TSL, by stabilizing the grain boundaries [121]. Using intravital microscopy, we showed that an optimized TSL composed of DPPC:DSPC:mPEG2000-DSPE (85:10:5 mol%) delivered an over 30-fold higher amount of DXR into the heated tumor compared to administration of free DXR [24,122]. Our studies suggest that DPPC and DSPC contents of 75–85 mol% and 20–10 mol%, respectively, are optimal, while no cholesterol should be added to these TSL [26].

A great leap in the application of TSL happened when ultra-fast releasing PEGylated TSL containing lysolipid (LTSL) was invented [27,111,121,123,124] and the concept of intravascular drug release was introduced. Later, the group of Lars Lindner developed another ultrafast-releasing TSL, with a release rate comparable to LTSL at a mild HT of 42 °C, by addition of 1.2-dipalmitoyl-sn-glycero-3- phosphoglyceroglycerol (DPPGOG or DPPG2, T_c_ 39.7 °C), a derivative of the naturally occurring DPPG, into TSL composed of DPPC:DSPC [125,126], and which is now ready for clinical trial investigations under the commercial name of Thermosome^®^ (https://www.thermosome.com, accessed on 23 October 2010) [127].

**Figure 5:**
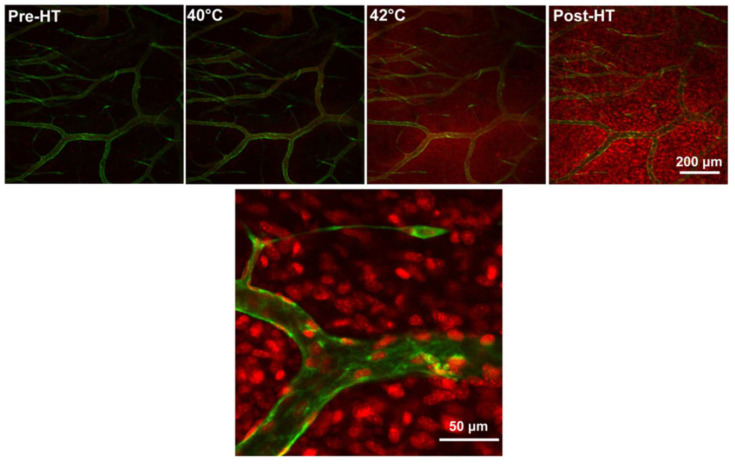
Heat-triggered DXR release from DXR-loaded TSL, and the subsequent intracellular nucleus delivery and accumulation in BFS-1 sarcoma cells and endothelial cells visualized in dorsal skin fold murine BFS-1 sarcoma. Reprinted with permission from [122].

In an intravascular drug release setting, a complete and fast drug release during the short transit time of TSL through the heated region (i.e., within seconds) is required, to create a steep concentration gradient between the blood and tumor interstitium [128] (Figure 4b). The magnitude of this gradient is the main factor that drives the diffusion of the released drug towards the tumor interstitium [49], making drug delivery independent of the EPR effect. In addition, the application of heat enhances the perfusion and permeability of the tumor vasculature, which not only augments the diffusion of free drug molecules, but also enhances the extravasation of TSL into the tumor. Therefore, intravascular drug release provides an opportunity to either bypass EPR-based drug delivery or enhance the EPR effect [110]. As can be seen in Figure 4c, intravascular drug release results in the massive diffusion and distribution of released contents into the tumor interstitium. Clearly, diffusion of the free small molecules takes place at a relatively fast rate, which, on the one hand, results in a rapid buildup of the released content in the tumor but, on the other hand, results in a rapid clearance from the tumor, unless the encapsulated content has specific affinity for the tumor tissue. This can be seen in Figure 5, where the released DXR reached the cell nuclei and stayed there, because of a covalent interaction with the DNA. Moreover, we observed that creation of a high concentration of DXR inside the tumor vasculature resulted in a massive nuclear delivery of DXR into the endothelial cells (Figure 5) [122], which can indirectly impact tumor growth through anti-vascular effects and could contribute, in part, to the reported improved anti-tumor response [122,129,130].

**Figure 6:**
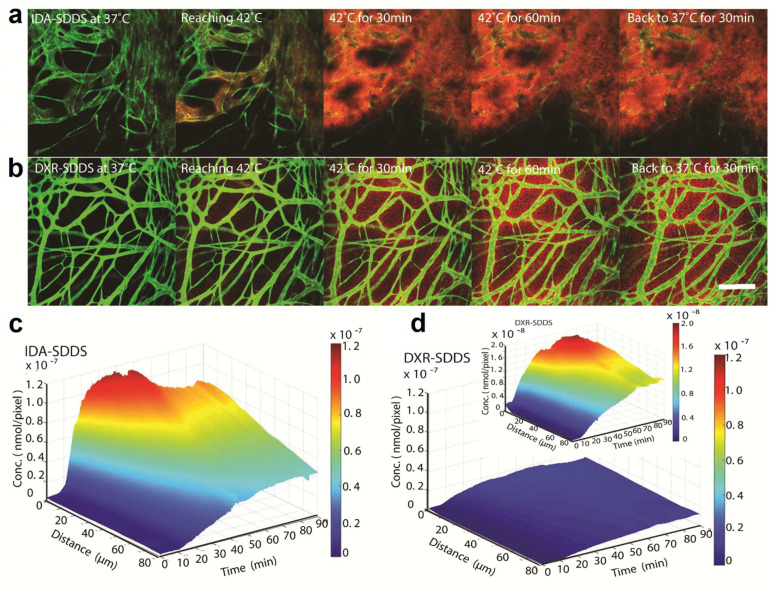
Rapid uptake of idarubicin (IDA) upon fast heat-triggered release from TSL results in a higher local drug delivery and a sharper intratumoral gradient compared to doxorubicin (DXR). (**a**,**b**) Imaging of the heat-triggered release in a window chamber fixed on eNOS-Tag-GFP mice with green vessels. Eighteen µmol/kg of IDA-SDDS (**a**) or DXR-SDDS (**b**) was injected, followed by 1 h local hyperthermia and 30 min normothermia, showing the intravascular drug (red) release and the subsequent diffusion into the tumor interstitium. Scale bar, 200 µm. Panels c and d show the 3-D representation of intratumoral drug concentration, as a function of time and distance from the nearest vessel. The faster and higher uptake of IDA (**c**) than DXR (**d**) by the tumor is clear (*n* = 3 mice per group). Reprinted from [28] under the Creative Commons license.

It is important to take into account that, to realize all the benefits of combining heat with TSL, the application of heat and administration of TSL need to take place concomitantly. Conversely, the target temperature should be reached before injection of TSL. Generally, the combination of heat and TSL can be done in three modes, with respect to the timing of dosing and heating. Other than intravascular drug release, in another approach, one can inject TSL and wait until the maximum accumulation of liposomes has been built up in tumor and then apply heat to trigger the release extravascularly. In this strategy, which is quite common in preclinical applications of heat sensitive drug delivery systems, none of the advantages of heat in increasing the drug delivery to tumor are employed. Accumulation of liposomes relies solely on the EPR effect, but the encapsulated drug can become bioavailable inside the tumor or inside tumor cells. As stated above, for intravascular drug release, the capability for fast drug release is crucial, which is, however, often accompanied by intrinsically premature drug leakage and a fast clearance. To overcome these limitations and take some advantage of the heat application, we and others have proposed a third mode, where a so-called two-step mild hyperthermia approach could be applied [25]. First, a mild hyperthermia cycle (e.g., 30 min to 1 h) could be applied prior to TSL administration, to induce the biological effects of heat on the tumor and tumor cells. After the first HT cycle, when tumor has returned to body temperature, but the elevated vascular perfusion and permeability inside tumor have not yet returned to the ground state (e.g., 1 h after heat), TSL could be administered. Finally, a second heat cycle could be applied when the accumulation of liposomes inside the tumor is at the maximum level. In this mode, a fast drug release is not a prerequisite, and more essential is the liposome longevity in the blood. Therefore, more stable liposomes, with a longer circulation time but slower release rate, could be used, while the EPR-based accumulation is enhanced due to the first cycle of heat. However, it has been shown that, compared to intravascular drug release, this approach may be less effective [25]. Recently, Al-Jamal et al. [131] reported the same results and quantitatively showed that the heat-trigger-released drug can be washed away by diffusing into the blood stream upon application of the second heat. Theoretically, this could be expanded to the extravascular drug release. Considering the fact that extravasation of liposomes mostly happens in the well vascularized tumor periphery and liposomes mostly remain in the perivascular regions, upon release, the concentration gradient directs the drug diffusion towards the sink condition of the blood flow. Despite the proven superior performance of intravascular release compared to the other modes, we have seen such a backward diffusion of DXR from the tumor interstitium into the blood flow upon stopping the heat, which was observed as a decline in the drug levels in the extracellular regions [122]. This indicates that not all the DXR successfully delivered to the tumor tissue can successfully interact with the target, the tumor cell, most likely due to saturation or slow cellular uptake. We have recently developed an in vivo derived computational model, to simulate intravascular drug delivery by TSL [113]. By using data collected from two TSL formulations with slow and fast drug release rates, this model identified parameters related to the kinetics of release from TSL, physicochemical properties of drug, and the target tissue for optimizing the intravascular drug release. Our model indicates that a fast drug release (in few seconds) and a drug with rapid tissue uptake (i.e., high first-pass extraction fraction) are the most important parameters that dictate the overall drug delivery to the tumor. We have shown in vitro and in vivo that replacing DXR with idarubicin, a less hydrophilic anthracycline, resulted in greater drug uptake and a better anti-tumor response [28,51], which could be attributed to the faster and greater uptake of idarubicin (Figure 6).

One serious issue that still needs to be resolved is the premature release from fast releasing TSL, which causes a fast clearance rate and thus an impaired delivery to tumor cells. Increasing the release rate with mild hyperthermia is intrinsically accompanied with an increased leakage at 37 °C. It has been shown that, in patients receiving an infusion of 50 mg/m^2^ DXR in LTSL, the DXR level in plasma 1 h after reaching C_max_ is almost half of the C_max_; indicating that during 1 h of hyperthermia, applied 1 h after reaching the C_max_, the doxorubicin availability in tumor vessels had dropped twofold. It is worth mentioning that, at C_max_, only 58% of plasma DXR is liposomal encapsulated and 42% of the encapsulated DXR has already been released [132]. This could, in part, have contributed to the recent failure of LTSL in a phase III clinical trial (OPTIMA) [133].

While, in LTSL, the presence of 5 mol% mPEG2000-DSPE is beneficial for enhancing the circulation time and release rate, compared to non-PEGylated liposomes, DPPG2 has been described as a promising alternative to PEG, which increases TSL circulation half-life and facilitates rapid drug release under mild hyperthermia. However, despite the success of replacing PEG with DPPG2, in reducing the PEG-related immunological reaction and accelerating blood clearance upon repeated injections, it cannot increase blood circulation longevity comparably to 5 mol% of PEG2000 [134]. It was shown that in pigs receiving 60 mg/m^2^ DXR in DPPG2-TSL, the concentration of DXR 30 min after reaching C_max_ was 42% of the C_max_ [135]. Therefore, no significant improvement with respect to DXR circulation life time compared to LTSL is expected. In principle, during the course of hyperthermia, the availability of liposomal drug greatly declines over time, which significantly hampers drug delivery and raises concerns about the efficacy of these formulations.

Our computational model supports the importance of a fast and complete drug release from TSL, to achieve an effective drug delivery to the tumor. However, in our model, it was assumed that the concentration of liposomal drug remained almost unchanged during the course of hyperthermia, and there have been no detailed studies that have correlated the PK behavior of TSL with the release kinetics and drug delivery to the tumor. It has to be mentioned that the DXR-loaded TSL composed of DPPC:DSPC:mPEG2000-DSPE (80:15:5 mol%) developed in our lab [24,122] are less leaky (less than 8% release during 1 h incubation at 37 °C in serum) than LTSL at 37 °C, but they cannot release as fast as LTSL at 42 °C. Interestingly, this preparation was found to be more effective in the treatment of a mouse model of B16 tumor compared to LTSL [122]; most likely due to a longer circulation time, which likely compensated for the incomplete release during one passage through the heated tumor. This observation highlights the importance of the circulation longevity of TSL. We believe that more attention has to be paid to the reformulating of TSL for increasing the circulation time.

## 5. Active-Targeted Drug Delivery in Cancer Therapy

Liposomes are considered successful nanocarriers, due to the potential simultaneous delivery of hydrophilic and hydrophobic drugs, owing to the improved pharmacokinetics and reduced off-targeted cytotoxicity associated with administration of free chemotherapeutic molecules. In fact, the evolution of liposomes from preclinical studies to clinical use is indebted to their capability for improving pharmacokinetics of chemotherapeutics, by limiting the distribution volume to the blood pool, which reduces the unwanted distribution of drug to healthy tissue and elongates the circulation in the blood, which, in turn, enables liposomes to accumulate in the tumor over time, based on passive targeting. Although the design of nanosized, stealthy, and stable liposomes guarantees a higher drug delivery to tumors compared to the free form [136,137], the cellular drug delivery and availability is negatively impacted by the steric hindrance effect of surface-grafted PEG and the lack or limited degree of drug release from the liposomes composed of rigid membrane. It is now well accepted that patients mostly benefit from reduced side effects, rather than an improved anti-tumor response, when treated with nanocarriers, in comparison to the administration of the free form of chemotherapeutics [138,139].

To enhance cellular drug delivery, one approach is to direct the liposome entry into cells via receptor-mediated endocytosis, which is also called active targeting. It should be noted that the term “active targeting” is generally used to refer to the application of ligand modified carriers, but this may mislead readers as signifying a smart drug delivery system that preferentially accumulates inside a tumor due to an additional functionality, such as specific ligands. It should be taken into account that, when targeting cells inside the tumor is the aim, the main mechanism of local delivery is passive tumor accumulation, where functionalized nanocarriers are able to actively interact with the targeted receptor on the targeted cells. Similarly to many other research groups, here at the NICE, different kinds of moieties for different active targeting approaches have been employed, to enhance the cellular internalization of liposomes to the target cell.

### 5.1. Anti-Vascular Targeting

**Figure 7:**
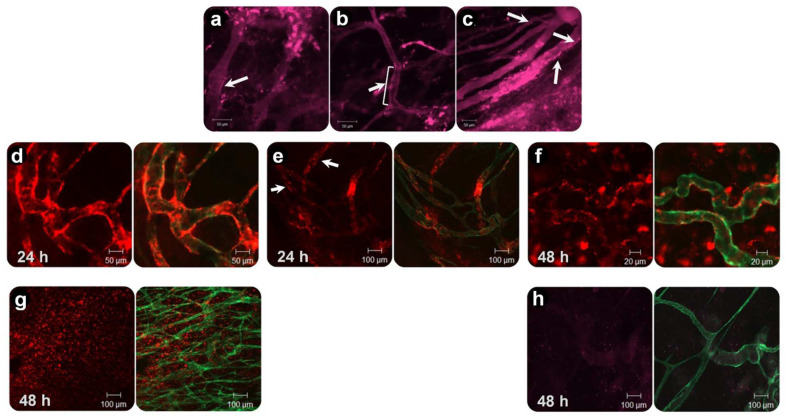
Intratumoral behavior of DiD-labeled RGDf[N-met]C-liposomes in NMRI nu/nu mice bearing BLM tumor (**a**–**c**), and Rho-PE-labeled (red) RGDf[N-met]C-liposomes in C57 mice bearing B16F0 tumor (**d**–**g**) imaged by intravital microscopy, using dorsal skin-fold chamber. Panel (**h**) illustrates the intratumoral behavior of DiD-labeled (purple) plain liposomes. RGDf[N-met]C-liposomes were found to be associated with the tumor vasculature (arrows in **a**–**f**) or extravasated and associated with tumor cells and likely other cells in the tumor region (**g**). Meanwhile, few plain liposomes were found associated with cells inside or outside the tumor vasculatures. Reprinted with permission from [29].

In 1984, Denekamp [140] proposed that by destruction of the existing tumor vasculatures, a large number of tumor cells can be impacted by being deprived of an oxygen and nutrition supply. An important advantage of anti-vascular targeting over targeting cells inside the tumor (tumor supporting cells or the malignant cells) is the direct accessibility of vascular endothelial cells for liposomes circulating in blood [141]. Thus, vascular-targeted liposomes can reach the target site, without relying on EPR. In other words, vascular targeting of nanomedicine is an alternative approach for bypassing the EPR. In addition, non-malignant tumor endothelial cells are genetically more stable and, therefore, are less prone to developing drug resistance or to showing downregulation of the targeted receptor [142]. Furthermore, vascular-targeted nanoparticles could be used against several kinds of cancer [141,142,143]. Among the numerous vascular-specific markers that are absent or lowly expressed in quiescent vessels, but that are overexpressed in the tumor vasculature (as reviewed by [144,145,146]), integrins, particularly αvβ3, αvβ5, and α5β1, are some of the most studied. The small sequence of amino acids Arginine, Glycine, and Aspartate (RGD) is a repeating unit in proteins that can interact with 24 members of the integrin family, known as the universal binding site of Fibronectin [147]. By adjusting the amino acids flanking RGD, specificity and selectivity towards αvβ3, αvβ5, and α5β1, which are overexpressed in the tumor neovasculature, could be achieved [148,149,150,151]. However, similarly to many ligand-modified liposomal preparations, RGD-liposomes showed an enhanced clearance rate through a vast uptake by the reticuloendothelial system (RES), which could cause toxic effects in these organs. Following the pioneering studies of Schiffelers et al., and using a cyclic RGDfK peptide for modification of PEGylated liposomes, Amin et al. [152] found that a positive charge of the K residue negatively impacts the circulation lifetime of peptide-modified liposomes, while animals treated with RGDfK-modified liposomal doxorubicin (RGDfK-PLD, pegylated liposomal doxorubicin) showed a higher systemic toxicity compared to those treated with RGDyC or RGDf[N-Met]K-PLD. It was found that replacing K, Lysine with less hydrophilic amino acids, such as C, Cysteine, favored the liposome pharmacokinetics, while it did not impact on the effectiveness of liposomes decorated with these RGD peptides against cells expressing αvβ3 integrin. Another modification that improved the pharmacokinetics of RGD-modified liposomes was the introduction of N-Methylated amino acids into the cyclic RGD pentapeptides, in the position of K that also reduces the clearance rate of RGD-modified liposomes and improves selectivity towards αvβ3 integrin, thus improving the therapeutic efficacy of the RGD-modified liposomal doxorubicin. N-Methylation not only reduced the hydrophilicity of the peptide, but it also increased the cyclic peptide selectivity towards its receptor, by constraining the cyclic peptide conformation. Later, RGDf[N-Met]C peptide was designed and decorated on liposomes for vascular targeting [29]. While intravital imaging of liposome behavior showed promising vascular targeting in two, B16 and BLM, tumor models (Figure 7a–f), the presence of RGDf[N-Met]C did not cause a pronounced augmentation of the clearance rate compared to plain liposomes, which enabled the liposomes to extravasate passively and accumulate inside the tumor, where they were found to be associated with cells expressing αvβ3 integrin (Figure 7g). While the overall tumor accumulations of RGD-liposomes and plain liposomes were virtually identical, such enhanced intravascular and extravascular association of RGD-liposomes improved the anti-tumor activity of liposomal DXR against the B16 and C26 murine tumor models.

#### Combination of Anti-Vascular Targeting and Hyperthermia

As discussed earlier, for an efficient application of intravascular drug release, an ultrafast drug release during a short passage of TSL through the heated area is a prerequisite. We hypothesized that creation of an affinity between the tumor vasculature and TSL can cause a delay of the passage of TSL through tumor area, which enables slow release-TSL to be completely released inside the heated tumor vasculature. To evaluate this concept, two known vascular targeting agents were employed to create such an affinity on TSL.

- Cationic charged TSL:

Angiogenic endothelial cells overexpress negatively charged functional groups, which could be exploited for targeting of tumor vasculature via electrostatic interaction using cationic charged nanoparticles [153,154]. In addition, Bally and coworkers reported that certain proteins in the protein corona associated with cationic liposomes may facilitate their interaction with angiogenic endothelial cells [155].

**Figure 8:**
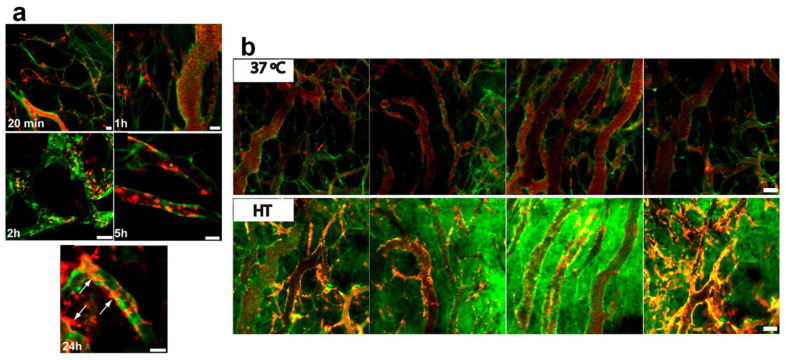
Intravital evaluation of the binding and temperature triggered release of CTSL. Panel (**a**) represents the binding of Rho-PE-labeled (red) CTSL to tumor endothelial cells (green). Appearance of vasculature-bound cationic liposomes started 20 min after injection and remained visible for at least 24 h. CTSL were found to be associated with the tumor vasculature or extravasated perivascularly (white arrows). Scale bar for all images indicates 20 μm. Panel (**b**) illustrates the release of carboxyfluorescein (CF) from CTSL upon application of HT. Images of circulating liposomes in bloodstream at 37 °C were taken 2 h after injection. Then, the tumor was heated up to 43 °C for 1 h, and representative images from different positions in the tumor area were recorded at the end of the HT treatment. Scale bar in all images, 50 μm. In both studies, C57 mice bearing B16BL6 tumor models, using a dorsal skin-fold chamber, received a single IV dose of 5 μmol liposomal lipid. Reprinted with permission from [31] Copyright 2013, American Chemical Society.

We observed that, while TSL (ζ potential −11 ± 0.7) composed of DPPC, DSPC and DSPE-PEG2000 (70:25:5 mol%) exhibited a Tc of 44.3 °C, replacing 10 mol % of DPPC with DPTAP in cationic charged TSL (CTSL, ζ potential 13 ± 1.0) increased the Tc to 47.4 °C, which resulted in temperatures of maximum release of 43 °C and 45 °C, respectively [31]. However, the magnitude of drug release from both liposomes at 37 °C and 42 °C after 5 min incubation in 90% serum was virtually identical. CTSL released about 5% more of the encapsulated CF than the TSL at both temperatures. In vitro studies showed that the addition of the cationic lipid accelerated the cellular association and internalization of liposomes into both BLM melanoma and normal HUVEC cells, and the application of heat upon internalization of liposomes resulted in intracellular release. Monitoring the in vivo behavior of CTSL and TSL, labeled with Rho-PE lipid, using intravital confocal microscopy in two, B16BL6 and LLC, tumors revealed the formation of patchy clusters of immobile red fluorescence in tumor vasculatures, indicating the association of liposomes with tumor vasculature (Figure 8a). This happened a few minutes after administration of CTSL, whereas no significant association between TSL and the tumor vasculature was observed. In addition, at later time points after the injection, the CTSL were also found to be extravasated, mostly in the perivascular regions and associated with cells inside tumors (Figure 8a). When CTL loaded with CF was administered to animals, no significant release of CF in the blood was observed before the application of heat. However, when 2 h of heat (43 °C) was applied, a remarkable cellular association and release of CF were observed (Figure 8b).

The study successfully visualized the combination of anti-vascular targeting and heat triggered drug release. Following this study, the therapeutic potential of such a system was investigated using DXR-loaded cationic liposomes [54]. First, it was observed that incorporation of 10 mol% DPTAP resulted in colloidal instability, after remote loading with DXR. Additionally, it also hampered the heat triggered release rate of DXR. These problems were resolved by reduction of DPTAP to 7.5 mol%. Compared to DXR-TSL, DXR-CTSL exhibited a greater cellular uptake and toxicity against different malignant and normal cell lines. When DXR-CTSL was combined with heat in vivo, it delivered a three-times higher amount of DXR to the tumor compared to DXR-TSL. In addition, this combination resulted in significant damages to the tumor vasculature. It was found that the presence of a positive charge did not make a significant impact on the clearance rate of TSL. Notably, both preparations exhibited a much shorter circulation time (~50% decline from C_max_ within 1 h) compared to non-thermosensitive liposomal DXR [32]. Therapeutic efficacy studies were performed in two different settings of extravascular release and with two-step mild hyperthermia. In one-step extravascular drug release, B16BL6 tumor model mice received 3 mg/kg liposomal DXR, and 5 h later, the tumor was heated to 42 °C for 1 h. No significant difference in the tumor growth rate of mice treated with DXR-TSL and DXR-CTSL was observed. Then, in a two-step mild hyperthermia, xenografted LLC tumor was initially heated (41 °C) for 1 h, to accelerate liposomes extravasation, and then, after 15 min of cooling down, liposomal DXR was injected, and 5 h later, a HT of 42 °C was applied for 1 h. The two-step mild hyperthermia was found to be more effective than the one-step extravascular release approach, and CTSL was more effective than the TSL in slowing the growth rate of LLC tumor. Notably, the first heat resulted in a 1.7-fold higher amount of DXR in the tumor of mice who received CTSL compared to TSL, whereas in the one-step extravascular release, both liposomes created identical DXR levels in the B16BL6 tumor [32].

- RGD-modified TSL

Based on the same strategy, heat-triggered drug release from TSL was combined with vascular-targeted liposomes decorated with RGD peptide, using cRGDf[N-Met]C peptide [30]. The addition of peptide caused no significant impacts on the colloidal, morphological, and heat sensitivity of TSL, where both liposomes showed more than 90% DXR release during a 5 min incubation at 42 °C. In vitro flow cytometry and live cell imaging revealed a superior association of RGD-TSL with HUVEC, and to lesser extent with murine melanoma cell lines compared to plain-TSL. When injected into mice, the association of RGD-TSL with the tumor vasculature was detectable after 20 min and was observed up to 24 h. In contrast, no significant colocalization of plain-TSL with the tumor vasculature was observed. Both liposomal preparations exhibited a short circulation time; however, as was expected, RGD-TSL was cleared a little faster than plain-TSL from the blood. Despite this enhanced clearance rate, RGD-TSL delivered a higher amount of DXR into the tumor in a two-step mild hyperthermia setting.

### 5.2. Tumor Cell Targeting

**Figure 9:**
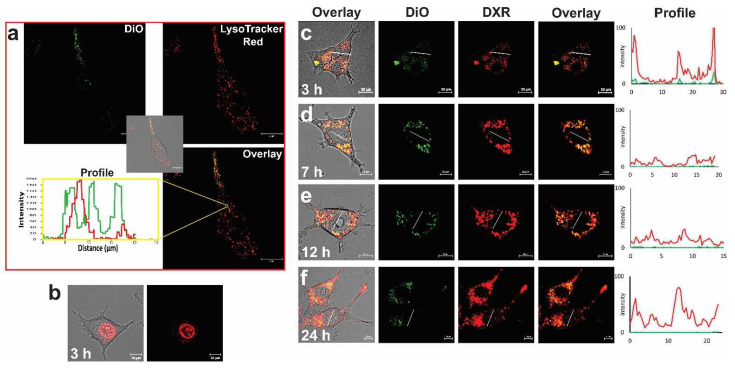
In vitro behavior of TAT-modified liposomes. Panel (**a**) illustrates internalization of Dio-labled TAT-modified liposomes by C26 cells via receptor-mediated endocytosis. Cells were pre-incubated with LysoTracker^®^Red, and the profile view of DiO (green line) and LysoTracker^®^ Red (red line) along a line passed through red and green spots inside the cell after 3 h of incubation at 37 °C is depicted. Panels (**b**–**f**) represent the intracellular fate of DXR delivered to C26 cells at 37 °C as free DXR (**b**) or encapsulated in TAT-modified DiO-labled liposomes (**c**–**f**) at different times, after 3 h of exposure to preparations and washing. Profile views represent the intensity of DiO (green line) and DXR (red line) along a line passed through the cell. Reprinted and modified from [33] under the Creative Commons license.

As stated earlier, one of the drawbacks of PEGylated liposomes composed of stable and rigid lipids, such as PEGylated liposomal doxorubicin (PLD), is the lack of a direct interaction with cells and the internalization of the liposomal cargo by cells, which hampers the cellular drug delivery. One solution to overcoming this is exploiting the distinct cellular markers that are overexpressed on malignant cells, by decorating nanoparticles with ligands that specifically interact with such receptors and are taken up by the cells of interest. Among the variety of ligands that have been introduced and studied for this purpose, a cell penetrating peptide derived from the transactivator of transcription of HIV-1 (TAT peptide) attracted our attention, to be used for promoting the cellular internalization of PLD. An analog of TAT peptide equipped with three Glycines (G) as spacer and a Cysteine (C) as conjugation functional group (CGGG-RKKRRQRRRGYG) was synthesized and conjugated to the distal end of PEG2000-DSPE and post-inserted into the outer surface of PLD at different densities (number of peptides/liposome) [33]. While post insertion of TAT-lipopeptide did not impact on the colloidal properties and stability of PLD, it only slightly reduced the negative zeta potential of the PLD, proportionally to the number of TAT. In vitro studies showed a direct correlation between the density of TAT peptide and the cellular association and internalization of liposomes. In contrast to Torchilin et al. [156], tracking the cellular uptake of TAT-modified liposomes using live-cell confocal microscopy demonstrated that TAT internalizes liposomes into cells via energy-dependent endocytosis (Figure 9a), which was consistent with some other studies [157].

Although the enhanced cellular association and avidity of PLD by TAT peptide was directly translated into enhanced cellular toxicity against different cell lines, we observed a delay in the delivery of DXR into the cell nucleus, in contrast to free DXR that rapidly reaches and interacts with the cell nucleus (Figure 9b). In other words, despite a massive uptake of TAT-PLD, only a limited amount of DXR was found in the cell nuclei, and which became detectable after several hours (Figure 9c–f).

**Figure 10:**
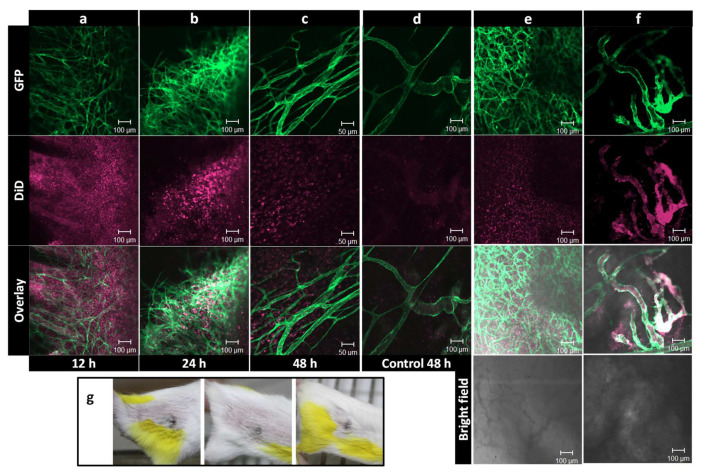
In vivo behavior of TAT-modified liposomes. Intratumoral behavior of DiD-labeled TAT-modified (**a**–**d**) or DiD-labeled plain liposomes (**e**,**f**) inside B16F0 tumors, using the dorsal skin-fold chamber upon i.v. administration of 5 μmol of liposomal lipid. While extravasated TAT-modified liposomes were found to be associated with tumor cells, few plain liposomes were observed as associated with cells inside the tumor. Panel (**g**) illustrates the manifestation of necrosis on the skin of dermal model of C-26 tumor followed by a single iv injection of 15 mg/kg liposomal DXR encapsulated in TAT-modified liposomes. Reprinted and modified from [33] under the Creative Commons license.

Due to the positive charge of TAT, we were concerned about the fast clearance of TAT-modified PLD. Therefore, in order to find an optimized preparation with the minimum required density of TAT that was effective while allowing liposomes to circulate for a long period, PLDs with different densities of TAT were tested in vivo for their biodistribution and therapeutic efficacy in a murine C26 tumor model. Through various biodistribution studies at different doses of liposomal DXR, it was found that, in contrast to our expectations, decoration of PLD with TAT did not enhance the uptake of liposomes by the liver and spleen; organs representing RES. Therefore, the PLDs with different densities of TAT (min 0 to max 200 peptide/liposome) had virtually the same circulation and were accumulated in the tumor to the same extent. To understand this, PLDs were further tested in vitro, with respect to the protein binding, by exposing liposomes at 37 °C. Interestingly, a direct correlation between the size of TAT-PLD and the TAT density was observed, when liposomes were exposed to mouse serum, while PDI remained unchanged. On the other hand, the zeta potential of all TAT-PLD became identical upon exposure to serum. These results imply that serum proteins are adsorbed on the surface of liposomes, to normalize the surface charge of liposomes, but this does not trigger aggregation, as was observed with RGD-modified liposomes [152]. It is likely that serum proteins such as albumin dysopsonize TAT-PLD by gently shielding the positive charge. This was also supported by a SDS-PAGE analysis of the protein corona associated with different PLDs. In accordance with this hypothesis, TAT-modified liposomes showed no affinity towards the tumor vasculature (Figure 10), as was observed with cationic liposomes. However, they were imaged as massively associated with cells inside the tumor after extravasation (Figure 10), which indicates that TAT residues retain their surface exposure and activity in the static condition of the tumor interstitium.

Therapeutic efficacy studies showed that around 100 TAT peptides per liposomes is an efficient density to improve the therapeutic efficacy of PLD. Interestingly, despite an identical accumulation of TAT-modified PLD in tumors, the most avid formulation decorated with 200 TAT peptides repeatedly exhibited the least effective preparation, while administration resulted in a significantly higher manifestation of skin necrosis at the site of the tumor (Figure 10g). Given the EPR-based accumulation, which mainly takes place in the tumor periphery, increasing the avidity negatively impacts the spatial distribution of liposomes, leaving the depth of tumor untouched, which resulted in a limited therapeutic effect, whereas the creation of a high concentration of DXR in the tumor periphery manifested in the occurrence of necrosis in tissues surrounding the tumor.

Other than TAT, Cetuximab (CTX, a monoclonal anti body (mAb) against epidermal growth factor receptor (EGFR)) was also used to target PEGylated liposomes loaded with Oxaliplatin at EGFR-expressing colorectal cancer [34]. Both whole mAb (CTX) and Fab′ fragments where conjugated on the liposome surface. In vitro studies demonstrated that the cellular uptake of liposomes increases by increasing the density of the ligand, reaching a maximum of three-fold higher compared to non-targeted liposomes and, interestingly, sensitized Oxaliplatin-resistance cells to oxaliplatin by bypassing the resistance mechanism. The number of proteins required to reach the max cellular association was determined to be 10 for CTX and 31 for its Fab′ fragment. It was shown that the presence of CTX is less favorable for the longevity of liposomes in circulation, most likely due to exposure of Fc′ fragments to the RES, whereas liposomes modified with Fab′ fragment underwent a slower clearance, were accumulated to a higher extent in the tumor, and were more effective against a xenografted SW-480 tumor model.

### 5.3. Combination of Vascular Targeting and Tumor Cell Targeting

Although anti-vascular targeting is known as an alternative approach to bypassing the EPR effect, it has to be taken into account that a part of the vascular-targeted nanoparticles passively extravasate into the tumor interstitium, and if the targeted vascular marker is not expressed on tumor cells, that part of the delivered particles behave the same as a non-targeted preparation. Importantly, the expression of such vascular markers, particularly αvβ3 and αvβ5 integrins, is dependent on the kind and stage of tumor and even the location of vessels [158], which makes anti-vascular targeting heterogeneous.

Moreover, the expression of targeted receptors on tumor malignant cells is also heterogeneous, and since active targeting to cells residing inside a tumor relies on EPR, such targeting is impacted by the heterogeneous nature of the EPR [17,18,19]. This prompted us to combine both anti-vascular targeting using RGD peptide and tumor cell targeting using the TAT peptide in a dual-targeted liposome, in order to increase the tumor area where dual functional liposomes can independently interact with the tumor vasculature via RGD peptide, while circulating in blood and interacting with cells inside the tumor via the TAT residue upon extravasation into the tumor. To this end, dual modified PEGylated liposomes (DPL) were designed and prepared, and were compared against liposomes modified with either TAT (TPL) or RGD (RPL), using the same peptides as used in our previous studies [35].

**Figure 11:**
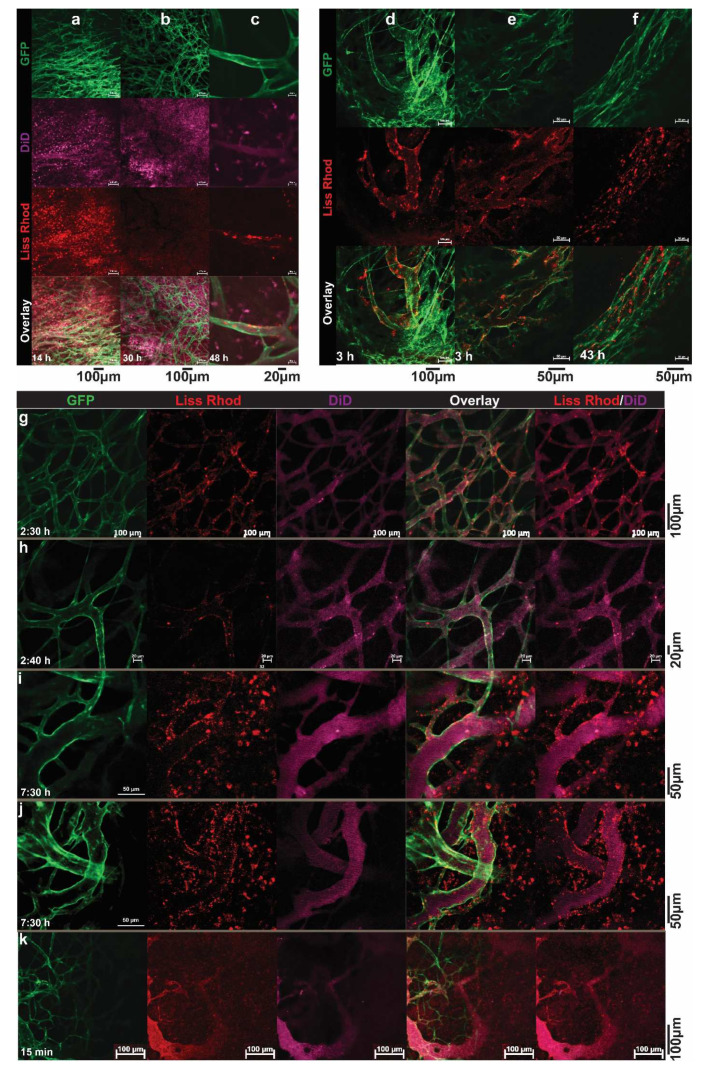
Intravital microscopy imaging of the intratumoral behavior of different ligand modified liposomes inside B16F0 tumors, using a dorsal skin-fold chamber. While in some regions both Rho-PE-labeled (red) RGD-liposomes and DiD-labeled (purple) TAT-liposomes exhibit almost identical cellular associations (**a**), in some regions, extravasated TAT-liposomes interact more than extravasated RGD-liposomes with cells (**b**), while RGD-liposome can be seen to be associated with tumor vasculature (**c**). Panels d-f illustrate the intratumoral behavior of Rho-PE-labeled (red) dually targeted liposomes in targeting tumor vasculature (**d**,**e**) or extravascular association with cells (**f**). When dual-liposomes (red) were co-injected with RGD-liposomes (purple), in most captured images (**g**–**j**) dual-liposomes exhibited superior vascular targeting compared to RGD-liposomes, which in some regions failed to interact with tumor vessels (**i**,**j**). Image (**k**) shows the cellular association of dual-liposomes, while RGD-liposomes did not interact with cells inside tumor interstitium upon extravasation. Mice were injected with 5 μmol of each liposomal lipid and images were captured at different time points after injection. Green fluorescence of endothelial cells is due to eNOStag-GFP expression. Reprinted and modified from [35] under the Creative Commons license.

The intratumoral behavior of these liposomes was compared by intravital microscopy upon i.v. injection of a cocktail of pairs of these preparations. Concomitant administration of TPL and RPL revealed a predictable functionality of TPL inside the tumor, while an association with the tumor vasculature was not observed (Figure 11a–c). In contrast, although RPL could be seen to be associated with both the tumor vasculature and tumor cells in some parts of the tumor, the targeting appeared heterogeneous. Not all tumor vasculature was targeted, and in some regions, despite extravasation of a significant amount of RPL, no cellular association was observed (Figure 11a–c). In contrast, DPL exhibited a remarkable vascular association (Figure 11d,e), and when extravasated into the tumor interstitium, it was always found to interact with cells (Figure 11f). When a cocktail of DPL and RPL was injected, it was observed that DPL dominated the vascular targeting and showed superior co-localization with tumor vasculature, compared to RPL (Figure 11g–j). In addition, DPL was found to be more successful in interacting with the tumor cells (Figure 11i–k). Concomitant administration of DPL and TPL confirmed the extensive vascular targeting of DPL, while no pronounced co-localization of the TPL and tumor vasculature was found. However, inside the tumor interstitium, the cellular targeting with TPL was more pronounced. Intravital imaging clearly showed a shorter circulation time of DPL compared with other preparations. This was also confirmed in a quantitative biodistribution study of PLD modified with different peptides. Despite the faster clearance rate of DPL preparations, and consequently their lower levels in tumors, DPL containing 100 TAT peptides and 300 RGD peptides showed a superior anti-tumor activity in a mouse model of B16 tumor, after a single i.v. injection of 15 mg/kg liposomal doxorubicin compared to other peptide-modified preparations and plain-PLD. This could be attributed to the ability of DPL to deliver DXR to the tumor vasculature. However, with respect to the short circulation time and massive uptake in the liver and spleen, this system cannot be considered as a reliable and safe drug delivery system for toxic agents, while it could be promising when used for vascular delivery of non-toxic or contrast agents. This study showed that the decoration of liposomes with two ligands for different sites in a tumor could increase the targeting area in the tumor and suggests that a combination of TAT and another vascular targeting agent could result in a better biodistribution profile [35].

## 6. Enhancing Cellular Drug Delivery by Short Chain Sphingolipids

Another approach to enhanc cellular drug delivery is the application of short-chain sphingolipids (SCS), which was exploited in the formulation of liposomes carrying chemotherapeutics.

It has been shown that the addition of ceramides to liposomes can increase the membrane permeability by induction of transbilayer motion [159,160]. In addition, self-association of ceramides in a membrane may result in the formation of domains or channels that can facilitate diffusion of amphiphilic drug molecules through the membrane [161]. Followed by studies of van Lummel et al. [36], different formulations of PEGylated liposomal doxorubicin enriched with different SCSs, including C8-glucosylceramide (C8-GluCer), C8-galactosylceramide (C8-GalCer), and C8-lactosylceramide (C8-LacCer), were formulated and extensively studied with respect to the formulation optimization and in vitro/in vivo drug delivery efficiency [37]. While C8-LacCer failed to form stable liposomes, the addition of up to 15 mol% of either C8-GalCer or C8-GluCer to the lipid mixture composed of HSPC: chol:mPEG-DSPE (62:33:5 mol%) resulted in liposomes around 100 nm, with a high remote loading efficiency (>90%), long-lasting storage stability at 4 °C, and minor leakage of around 10% during 24 h incubation at 37 °C in the presence of serum; comparable to liposomes without SCS. However, transmission electron microscopy revealed morphological changes in liposomes enriched with SCS upon loading with DXR. While both SCS-liposomes exhibited a normal spherical shape before loading, rod-shaped vesicles were found after DXR loading. Although elongation of liposomes due to the crystallization of DXR inside liposomes is a common observation, this was more pronounced in both SCS-containing preparations. Nevertheless, the addition of C8-GalCer resulted in more elongated liposomes (up to 500 nm) compared to the addition of C8-GluCer.

In vitro studies showed that while incorporation of SCS in DXR free liposomes has no impact on cell survival, it enhances the cellular delivery and toxicity of liposomal DXR, as was tested against MCF7 and SKBR3 breast carcinomas, BLM and Mel57 human melanomas, and Panc1 and ASPC1 pancreatic carcinomas. Addition of 10 mol% of SCS was found to be optimal, since above that, no further enhancement in cytotoxicity was observed. Importantly, minor, and no, enhanced toxicity was observed against normal fibroblasts (3T3) and HUVEC cells, respectively. This selective cellular delivery holds promise that SCS-enriched liposomal DXR does not have an impact on normal endothelial cells in healthy blood vessels. Live cell confocal imaging demonstrated that the cellular delivery of DXR was not associated with the uptake of NBD-PE-labeled SCS-liposomes, indicating that intracellular drug delivery by SCS is not dependent on liposome internalization. Later, it was found that spontaneous transfer of SCS from the liposome bilayer into the cell membrane, most likely the exoplasmatic leaflet, takes place rapidly upon cellular contact, during which the interaction and transmembrane transport of DXR are facilitated [38]. Recently, the capability of SCS-liposomal DXR in sensitizing the DXR resistance of tumor cells with an overexpressed level of Pgp was studied [39]. It was found that, while SCS-liposomes had no impact on the overexpression of Pgp by DXR-resistant human uterine sarcoma (MES-SA/MX2), it can reverse cell resistance, by modulation of cell membrane and drug uptake, rendering DXR as effective as in sensitive cells. Importantly, SCS-liposomes exhibited a superior net effect against a resistant cell line compared to the sensitive variant (MES-SA). This effect was translated into a greater anti-tumor effect of SCS-liposomal DXR against xenografted MES-SA/MX2 tumor model compared to the MES-SA sensitive tumor counterpart.

In addition to DXR, liposomal preparations enriched with SCS were also exploited for loading of Mitoxantrone (MTO), an anthracenedione antineoplastic antibiotic that is used in the treatment of acute leukemia, lymphoma, prostate, and breast cancer [40,52]. Similarly to DXR, MTO also needs to reach the cell nucleus to condensate DNA and inhibits replication and RNA transcription. When an ammonium sulfate gradient was employed for remote loading of liposomes (the same lipid composition as used for DXR loaded CSC-liposomes) incorporation of SCS (C8-GalCer and C8-GluCer) improved the loading efficiency, reaching a 100% drug/phospholipid ratio (*w*/*w*) of 0.07, whereas in the absence of CSC, the maximum obtained loading efficiency was 75%. It is likely that the interaction and hydrogen binding of MTO molecules with hydroxyl groups of the sugar moiety of the SCS head group enhances the MTO passage through the liposomal bilayer [39]. However, despite a stable colloidal property, both SCS-enriched liposomes and SCS-free preparation exhibited 6–19% MTO release during a week of incubation at 4 °C, and when exposed to 50% human serum at 37 °C, a burst release of 20% during the first hour, followed by slow gradual release up to another 10% after 24 h, was observed. Unlike DXR-liposomes, SCS-liposomes did not undergo morphological changes after drug loading and stayed spherical. Compared to normal liposomes, the in vitro cellular delivery of MTO into SKBR-3 breast carcinomas was improved by 12 to 15-fold and three-fold with C8-GalCer-liposomes and C8-GluCer-liposomes, respectively. Meanwhile, no pronounced cellular delivery into normal HUVEC and 3T3 cells was observed. The difference in MTO delivery by C8-GalCer-liposomes and C8-GluCer-liposomes into same cell line could be attributed to their equatorial and axial differences, where the orientation of the hydroxyl group at the 4th carbon atom is different, which may have differently impacted the lipid rearrangement and packing. Live cell imaging of liposomes double labeled with NBD-GalCer and/or Rho-PE revealed that the intracellular delivery of MTO by SCS-enriched preparations is independent of liposome uptake.

Analyzes of snap-frozen xenografted MCF-7 tumor tissue isolated 24 h, after injection of either free or liposomal MTO (5 mg/kg), indicated improved drug delivery by liposomes. MTO was only observed in the tumors of mice treated with liposomal preparations. Importantly, while only a marginal nuclear delivery located at the tumor periphery was observed following administration of free drug, the MTO fluorescence after administration of liposomal preparations was observed in more deep regions of the tumor. Both SCS-liposomal MTO formulations were further evaluated in vivo, with respect to PK and the anti-tumor activity in a mouse model of MDAMB-231 breast carcinoma [40]. At a single dose of 5 mg/kg MTO, due to the wide distribution throughout the body and fast clearance rate of free drug, MTO was not detectable in the blood at selected time points after administration of free MTO. Liposomal preparations circulated for longer. However, both SCS-liposomes exhibited shorter circulation times compared to the SCS-free liposome. The AUC of C8-GluCer-liposomes and C8-GalCer-liposomes were two- and three-times smaller than the AUC of control liposomes. Higher levels of MTO in the kidneys of mice receiving SCS-liposomal MTO is an indication of MTO leakage from these preparations. Meanwhile, C8-GalCer-liposomes were found to be leakier than C8-GluCer-liposomes, which correlated with their different AUC values. However, intravital imaging revealed a much faster intratumoral MTO fluorescence after administration of both SCS-liposomes compared to the control liposomes, which indicates that enrichment of liposomes with SCS enhances the intratumoral bioavailability of MTO. This enhanced availability of MTO was reflected by a better anti-tumor activity of SCS-liposomal MTO, especially for C8-GluCer-liposomes against an MDAMB-231 breast carcinoma tumor model, upon multiple injection of five doses of 5 mg/kg MTO, at weekly intervals.

## 7. Liposome-Based Cancer Immunotherapy

Cancer immunotherapy, which refers to harnessing the host immune system, either towards inhibition or stimulation of specific immune responses against cancer or the regulation and manipulation of the immune system against cancer cells, has become the most promising approach in the treatment of cancer, with impressive progress during the last three decades. Understanding tumor immunology has revealed mechanisms and factors involved in the escape of cancer cells from immune surveillance and showed how cancer cells harness immune responses in favor of their proliferation. Exploring the role of factors such as immune checkpoints and immunosuppressive cytokines, as well as the downregulation of immune stimulatory biomolecules, along with the emergence of chronic inflammation that results in immune dysfunction and interferences with the intrinsic activity of the host immune cells, provides opportunities to help the immune system to attack malignant cells [162,163]. Nanocarriers, especially liposomes, have been exploited in immunotherapy, either as carriers or adjuvants in cancer vaccines [164], or for selective delivery of immunomodultory agents [165,166,167]. In fact, liposomes might be able to overcome several major drawbacks faced by cancer immunotherapies through development of a potential platform for delivery of stimulatory ligands (immunostimulatory adjuvants, immunogenes, immunostimulatory molecules), for modulating immune responses, immune checkpoint blockade molecules (CTLA-4, PD-1, PD-L1), small molecules (indoleamine 2,3-ioxygenase, TGF-β, adenosine, and IL-10) to target the modulation of the tumor microenvironment, and combinational therapy. Therefore, different liposomal-based platforms have been explicitly developed for cancer immunotherapy [165,166,167].

Melanoma is a good example of the development of immunotherapy. While improved clinical outcomes are apparent, quite a few patients fail to respond from the beginning, while others develop resistance over time. We and others have investigated specific melanoma targeting [41,42,168] and the complexation of checkpoint inhibitors [43,169] mediated by lipid-based nanocarriers, to provide a means to improve responses and to engage in combination therapy. To target melanoma and to develop a platform that can be used to either perform immunotherapy or targeted chemotherapy, or combine these two approaches, we studied the possibility of using antibody-conjugated liposomes. To do so we generated an antibody, single chain variable fragments (scFv), designated G8 [41]. This scFv targets melanoma the antigen MAGE A1 when presented by HLA-A1. The concept is that this antibody acts like a T-cell receptor (TCR) and, as such, increases the specificity of the antibody for melanoma cells. Indeed, specific recognition is achieved. It is important to note that different antibodies were used, and the affinity of the improved antibody version, designated Hyb3, while possibly showing improved binding to cells, also showed an enhanced recognition of cells not positive for MAGE-A1. Comparably, to improve binding by using affinity matured antibodies, antibodies were generated for MAGE-A1 peptides, by using MAGE-A1 peptides with an augmented affinity for HLA-A1 [168]. These so-called heteroclitic peptides are changed by insertion of certain amino acids, and by doing so have improved binding to HLA. However, this change can alter exposure of the right antibody recognition site and thus generate antibodies that will not recognize the wild type peptide. Clearly, one must protect the binding specificity when aiming for improved affinity. While these liposomes could be used for immunotherapy, the efficacy of targeting solid tumors in mice was first studied in a chemotherapeutic setting. Stable Doxil-like liposomes decorated with either G8 or Hyb3 were injected in melanoma-bearing mice [42].

To demonstrate specificity for melanoma cells and, moreover, to demonstrate specificity for melanoma cells expressing MAGE-A1 in a HLA-A1 context, mice were installed with melanoma tumors positive for HLA-A1 but with, or without, expression of MAGE-A1. Doxorubicin-containing liposomes decorated with G8 specifically caused an improved anti-tumor effect in MAGE-A1+/HLA-A1+ tumors, while Hyb3 decorated liposomes induced a response in both MAGE-A1-positive and -negative tumors. These results corroborated the binding studies and are an indication that specificity and affinity may not always go hand-in-hand.

## 8. A NICE Perspective

The nanomedicine field has developed fast and an increasing number of basic, translational, and clinical researchers, from a plethora of disciplines, have become involved. Already, a vast number of departments, research groups, and laboratories with nanomedicine, or something alike, in their name are interested in the application of nanotechnology for advanced treatment of patients.

Our experience started with a relatively simple and, above all, practical approach. Patients with locally progressed disease, i.e., advanced melanoma or sarcoma in the arm or leg and demanding amputation, profited enormously from a straightforward clinical approach. Isolation of the diseased area resulted in a reduction in the volume of distribution and protected sensitive tissues from exposure to the locally administered chemotherapeutic. This resulted in an increase in local drug concentration and a favorable tumor response in most of these patients. The realization that delivery matters was an eye opener. More so, these results implied that maybe delivery is key, rather than drug resistance.

To improve delivery and curb systemic toxicity, liposomal formulations were developed, resulting in long circulating PEGylated liposomal doxorubicin. Doxil, the first registered liposomal chemotherapeutic, indeed altered the toxicity profile; and especially in animals, elevated accumulation of drug in tumors was observed. In most clinical applications this formulation appeared to not be more effective than free doxorubicin. With the aid of intravital microscopy, an invaluable tool, especially in a setting in which the kinetics of carrier and drug in a tumor needs to be studied in real time, we observed that doxorubicin was only slowly separated from the liposomes, causing the levels of available drug to be lower than expected. For many applications of nanomedicine formulated for systemic (i.e., intravenous or intra-arterial infusion) administration, the stability of the formulation in circulation conflicts with the need for the drug to become available at the site of interest. This mismatch and the misconception that intracellular nanoparticles will be degraded, and thus the drug will be released and will be available for action, are still affecting a significant number of studies performed today.

With the realization that nanoparticles may be too stable, several groups have developed liposomes that can be ordered to release their contents. We optimized so-called thermosensitive liposomes (TSLs) that have the benefit of a long circulation at 37 °C (i.e., prolonged presence of drug in the circulation, diminished degradation, and diminished side-effects), and which allow a triggered release of their content at the tumor site. This approach has the potential to act as a loco-regional treatment setting, with the potential to massively improve drug accumulation. Owing to our background in loco-regional treatment, two aspects of this approach surprised us. First, we asked whether the drug mostly used, doxorubicin, was indeed the most ideal. Second, loco-regional treatment of cancer is mostly used to achieve local control, and not to obtain a prolonged survival or cure per se.

As mentioned above, we questioned whether doxorubicin was indeed a good choice to be used in TSLs. We showed through intravital microscopy that, after cessation of hyperthermia, the trigger applied to force release form TSLs, interstitial drug levels dropped sharply, causing a significant part of doxorubicin to sink back into the circulation. Interestingly, the drug levels reached in the tumor were such that cellular uptake was the rate limiting step. If this approach is aimed at obtaining locally an optimal effect, a drug with more favorable characteristics, fitting such approach, should be used. We identified idarubicin and, indeed, observed that this drug, when used in a TSL setting, inflicted more harm to the tumor because of the faster uptake of the drug by tumor cells. However, while this could be better for targeted treatment of a tumor, such a drug would have little effect on metastasis, because of the short circulation time in the free form.

The TSL-based approach, in combination with hyperthermia, is often challenged by the fact that patients mostly die because of metastases. Thus, targeting the individual tumor does not really help to improve overall survival. While obtaining local control or rendering a tumor surgically removable is a not an achievement to be underestimated, and for a number of cancers this is dearly needed, improvements to survival or a cure are, of course, the ultimate goal. To understand the usefulness of nanomedicine and TSL-based cancer therapy, it is useful to realize that only a small fraction of the administered dose reaches the targeted tumor. In other words, most of the injected liposomes are available for targeting the metastases. Therefore, actually, the TSL plus hyperthermia approach is a systemic treatment where the local response is emphasized. This means that we have to generate liposomes that combine good stability in circulation with acceptable accumulation in metastases and distant tumors, while showing a fast response to the external trigger, followed by a fast uptake by tumor cells.

The future of liposomal chemotherapy and, for instance, the combination of hyperthermia with TSLs is certainly not set in stone, but also not chiseled. Recently the argument was made that delivery is key, and we agree that this is true. However, what is overlooked is the need for a better selection of the therapeutic. When considering hyperthermia with TSLs, an improvement, mostly a simplification of the heating method and application, is needed. This challenge will remain if we do not direct our focus and realize that it is the treatment of the individual tumor, combined with the spread of disease at the same time, that needs to be controlled. Our experience tells us that to continue this journey, we need the involvement of clinicians and, in particular, medical oncologists. We need to simplify our systems and adapt them to clinical practice. There are a multitude of different groups working on a range of different formulations, showing the capacity and possibilities nanomedicine provides. To refine these options, and to develop the best for the clinic, we need to identify the purpose of the formulations: is local control intended or patient well-being, is prolonged survival or a cure aimed for? The future looks promising, but also quite overwhelming; and to obtain a realistic and applicable formulation, we need to make choices and be modest about the endpoint we are aiming for. If we can do that, we certainly have a chance to better contribute to cancer therapy.

## 9. Conclusions

Since the first publications on liposomes and lipid nanoparticles by its founder, Alec Bangham, the field of nanomedicine has developed fast and resulted in the registration of Doxil/Caelyx. The literature on nanosystems for clinical application has accumulated fast, with more than 42,000 hits (PubMed) in 24 years for “nanomedicine” alone. This development clearly went through phases, and new inventions and disappointments followed each other. Liposomes became PEGylated to provide an improved circulation time and were formulated with rigid lipids, to provide robust and stable carrier, adding to the circulation and preservation of the payload. Soon after, it became apparent that such particles prevent delivery to the target, i.e., the tumor cell or tumor cell compartment. This initiated the development of trigger sensitive liposomes and the development of smart drug delivery systems (SDDS). Currently a plethora of nano-sized systems are being developed, researched, and tested, with sometimes exotic mechanisms or elaborate combination of substances and possibilities. However, as we argued above, clinical application is falling behind, and bringing more complex systems to the table is not helping to improve applicability in patients. Recent clinical trials with thermosensitive liposomes have indeed demonstrated that the combination of these formulations and heating technologies with the complexity of the patient and disease led to poor results. This is important, as pre-clinical studies and modelling indicated the potential of this approach. Our growing knowledge and what we learned from others has helped us to get to where we are now. We realize, however, that to progress further, i.e., move towards the clinic, we need to collaborate more than ever with all fields.

## Figures and Tables

**Table 1:** Different liposomal formulations studied at the Nanomedicine Innovation Center Erasmus (NICE).

Formulation	Composition mol:mol	Encapsulated Compound	Study Type	Cell Line	Tumor Model	Function	Ref
DPPC:DSPC: mPEG2000-DSPE	80: (20 − x): x (x = 1, 3, 4, 5, 6, 10)	CF	In vitro in vivo	BLM	BLM melanoma	TSL	[24]
DPPC:DSPC: mPEG2000-DSPE	55:40:5 80:15:5	DXR	In vitro in vivo	Different cell lines	BLM melanoma	TSL	[25]
DPPC:DSPC: mPEG2000-DSPE	x: (100 − x): 5 (x = 80, 70, 60, 50)	DXR	In vivo	-	BFS-1 sarcoma	TSL	[26]
DPPC:DSPC: mPEG2000-DSPE	x: (100 − x): 5 (x = 100, 80, 60, 40, 20, 0)	CF	In vitro	-	-	TSL	[27]
DPPC:DSPC: mPEG2000-DSPE	60:35:5 for IDA -TSL 70:25:5 for DXR -TSL	IDA DXR	In vitro in vivo	B16BL6, BLM, BFS-1	BLM melanoma	TSL	[28]
HSPC:Chol: mPEG2000-DSPE	56.1:38.2:5.5	DXR	In vitro in vivo	Different cell lines	C26 colon carcinoma B16F10 and BLM melanoma	Vascular targeting by RGD peptide	[29]
DPPC:DSPC: mPEG2000-DSPE	70:25:5	DXR	In vitro in vivo	B16BL6 and B16F10	B16BL6 melanoma	TSL + Vascular targeting by RGD peptide	[30]
DPPC:DSPC: DPTAP mPEG2000-DSPE	62.5:25:10:5	CF	In vitro in vivo	BLM and HUVEC	B16BL6 melanoma	TSL + Vascular targeting by positive charge	[31]
DPPC:DSPC: DPTAP: mPEG2000-DSPE	62.5:25:7.5:5	DXR	In vitro in vivo	B16 and LLC	B16BL6 melanoma	TSL + Vascular targeting by positive charge	[32]
HSPC:Chol: mPEG2000-DSPE	56.1:38.2:5.5	DXR	In vitro in vivo	Different cell lines	C26 colon carcinoma	Targeting tumor cells with TAT	[33]
HSPC:Chol: mPEG2000-DSPE: Mal-PEG2000 DSPE	1.85:1:0.12:0.03	Oxaliplatin	In vitro in vivo	Different cell lines	SW-480 colorectal cancer	Targeting tumor cells with Cetuximab	[34]
HSPC:Chol: mPEG2000-DSPE	56.1:38.2:5.5	DXR	In vitro in vivo	Different cell lines	C26 colon carcinoma B16F10 melanoma	Dual anti-vascular and tumor cell targeting with RGD and TAT peptides	[35]
HSPC:Chol: mPEG2000-DSPE: C8-glucosylceramide	1.85:1:0.15:15	DXR	In vitro in vivo	B16 cells A431	A431 epidermoid carcinoma	Short-chain sphingolipids	[36]
HSPC:Chol: mPEG2000-DSPE: C8-GluCer, C8-GalCer, C8-LacCer	1.85:1:0.15:15	DXR	In vitro	Different cell lines	-	Short-chain sphingolipids	[37]
HSPC:Chol: mPEG DSPE: C8-GlcCer, C8-GalCer	1.85:1:0.15:10	DXR	In vitro	Different cell lines	-	Short-chain sphingolipids	[38]
HSPC:Chol: mPEG2000-DSPE: C8-GluCer	1.85:1:0.15:10	DXR	In vitro in vivo	Different cell lines	MES-SA or MES-SA/MX2 uterine sarcoma	Short-chain sphingolipids	[39]
HSPC:Chol: mPEG2000-DSPE: C8-GlcCer, C8-GalCer	1.85:1:0.15:10	Mitoxantrone	In vivo	-	MDA-MB-231 and MCF-7 breast cancer	Short-chain sphingolipids	[40]
HSPC:Chol: mPEG2000-DSPE: maleimide mPEG2000-DSPE	55:40:4:1		In vitro	Different cell lines		Immunotherapy with Anti-MAGE A1 T-cell receptor (TCR)-like single-chain antibody	[41]
HSPC: Chol: mPEG2000-DSPE: maleimide mPEG2000-DSPE	55:40:4:1	DXR	In vitro in vivo	Different cell lines	G43 and Mel78 melanoma	Immunotherapy Anti-MAGE A1 TCR-like single-chain antibody	[42]
HSPC: Chol: mPEG2000-DSPE	1.85:1:0.12	DXR	In vitro in vivo	B16OVA murine melanoma	B16OVA B16OVA	Immunotherapy Monovalent-variable fragments (Fab’) of α-PD-L1	[43]

## References

[1] Bray F., Ferlay J., Soerjomataram I., Siegel R.L., Torre L.A., Jemal A. (2018). Global cancer statistics 2018: GLOBOCAN estimates of incidence and mortality worldwide for 36 cancers in 185 countries. CA Cancer J. Clin..

[2] Dickens E., Ahmed S. (2018). Principles of cancer treatment by chemotherapy. Surgery.

[3] Sharifi M., Cho W.C., Ansariesfahani A., Tarharoudi R., Malekisarvar H., Sari S., Bloukh S.H., Edis Z., Amin M., Gleghorn J.P. (2022). An Updated Review on EPR-Based Solid Tumor Targeting Nanocarriers for Cancer Treatment. Cancers.

[4] Kalepu S., Nekkanti V. (2015). Insoluble drug delivery strategies: Review of recent advances and business prospects. Acta Pharm. Sin. B.

[5] Papahadjopoulos D., Allen T.M., Gabizon A., Mayhew E., Matthay K., Huang S.K., Lee K.D., Woodle M.C., Lasic D.D., Redemann C. (1991). Sterically stabilized liposomes: Improvements in pharmacokinetics and antitumor therapeutic efficacy. Proc. Natl. Acad. Sci. USA.

[6] Thakor A.S., Gambhir S.S. (2013). Nanooncology: The future of cancer diagnosis and therapy. CA Cancer J. Clin..

[7] Wang A.Z., Langer R., Farokhzad O.C. (2012). Nanoparticle delivery of cancer drugs. Annu. Rev. Med..

[8] Douer D. (2016). Efficacy and Safety of Vincristine Sulfate Liposome Injection in the Treatment of Adult Acute Lymphocytic Leukemia. Oncologist.

[9] Deitcher O.R., Glaspy J., Gonzalez R., Sato T., Bedikian A.Y., Segarini K., Silverman J., Deitcher S.R. (2014). High-dose vincristine sulfate liposome injection (Marqibo) Is not associated with clinically meaningful hematologic toxicity. Clin. Lymphoma Myeloma Leuk..

[10] Silverman J.A., Deitcher S.R. (2013). Marqibo(R) (vincristine sulfate liposome injection) improves the pharmacokinetics and pharmacodynamics of vincristine. Cancer Chemother. Pharmacol..

[11] Passero F.C., Grapsa D., Syrigos K.N., Saif M.W. (2016). The safety and efficacy of Onivyde (irinotecan liposome injection) for the treatment of metastatic pancreatic cancer following gemcitabine-based therapy. Expert Rev. Anticancer Ther..

[12] Zhang H. (2016). Onivyde for the therapy of multiple solid tumors. Onco Targets Ther..

[13] Lamb Y.N., Scott L.J. (2017). Liposomal Irinotecan: A Review in Metastatic Pancreatic Adenocarcinoma. Drugs.

[14] Hare J.I., Lammers T., Ashford M.B., Puri S., Storm G., Barry S.T. (2017). Challenges and strategies in anti-cancer nanomedicine development: An industry perspective. Adv. Drug Deliv. Rev..

[15] Lammers T., Kiessling F., Ashford M., Hennink W., Crommelin D., Storm G. (2016). Cancer nanomedicine: Is targeting our target?. Nat. Rev. Mater..

[16] Matsumura Y., Maeda H. (1986). A new concept for macromolecular therapeutics in cancer chemotherapy: Mechanism of tumoritropic accumulation of proteins and the antitumor agent smancs. Cancer Res..

[17] Eberhard A., Kahlert S., Goede V., Hemmerlein B., Plate K.H., Augustin H.G. (2000). Heterogeneity of angiogenesis and blood vessel maturation in human tumors: Implications for antiangiogenic tumor therapies. Cancer Res..

[18] El Emir E., Qureshi U., Dearling J.L., Boxer G.M., Clatworthy I., Folarin A.A., Robson M.P., Nagl S., Konerding M.A., Pedley R.B. (2007). Predicting response to radioimmunotherapy from the tumor microenvironment of colorectal carcinomas. Cancer Res..

[19] Lee H., Shields A.F., Siegel B.A., Miller K.D., Krop I., Ma C.X., LoRusso P.M., Munster P.N., Campbell K., Gaddy D.F. (2017). (64)Cu-MM-302 Positron Emission Tomography Quantifies Variability of Enhanced Permeability and Retention of Nanoparticles in Relation to Treatment Response in Patients with Metastatic Breast Cancer. Clin. Cancer Res..

[20] Fang J., Islam W., Maeda H. (2020). Exploiting the dynamics of the EPR effect and strategies to improve the therapeutic effects of nanomedicines by using EPR effect enhancers. Adv. Drug Deliv. Rev..

[21] Shi Y., van der Meel R., Chen X., Lammers T. (2020). The EPR effect and beyond: Strategies to improve tumor targeting and cancer nanomedicine treatment efficacy. Theranostics.

[22] Golombek S.K., May J.N., Theek B., Appold L., Drude N., Kiessling F., Lammers T. (2018). Tumor targeting via EPR: Strategies to enhance patient responses. Adv. Drug Deliv. Rev..

[23] Gregoriadis G. (2018). Liposomology: Delivering the message. J. Liposome Res..

[24] Li L., ten Hagen T.L., Schipper D., Wijnberg T.M., van Rhoon G.C., Eggermont A.M., Lindner L.H., Koning G.A. (2010). Triggered content release from optimized stealth thermosensitive liposomes using mild hyperthermia. J. Control. Release.

[25] Li L., ten Hagen T.L., Haeri A., Soullie T., Scholten C., Seynhaeve A.L., Eggermont A.M., Koning G.A. (2014). A novel two-step mild hyperthermia for advanced liposomal chemotherapy. J. Control. Release.

[26] Lokerse W.J., Kneepkens E.C., ten Hagen T.L., Eggermont A.M., Grull H., Koning G.A. (2016). In depth study on thermosensitive liposomes: Optimizing formulations for tumor specific therapy and in vitro to in vivo relations. Biomaterials.

[27] Lu T., Ten Hagen T.L.M. (2017). Inhomogeneous crystal grain formation in DPPC-DSPC based thermosensitive liposomes determines content release kinetics. J. Control. Release.

[28] Lu T., Haemmerich D., Liu H., Seynhaeve A.L.B., van Rhoon G.C., Houtsmuller A.B., Ten Hagen T.L.M. (2021). Externally triggered smart drug delivery system encapsulating idarubicin shows superior kinetics and enhances tumoral drug uptake and response. Theranostics.

[29] Amin M., Mansourian M., Koning G.A., Badiee A., Jaafari M.R., Ten Hagen T.L. (2015). Development of a novel cyclic RGD peptide for multiple targeting approaches of liposomes to tumor region. J. Control. Release.

[30] Dicheva B.M., ten Hagen T.L.M., Seynhaeve A.L.B., Amin M., Eggermont A.M.M., Koning G.A. (2015). Enhanced Specificity and Drug Delivery in Tumors by cRGD—Anchoring Thermosensitive Liposomes. Pharm. Res.-Dordr.

[31] Dicheva B.M., ten Hagen T.L., Li L., Schipper D., Seynhaeve A.L., van Rhoon G.C., Eggermont A.M., Lindner L.H., Koning G.A. (2013). Cationic thermosensitive liposomes: A novel dual targeted heat-triggered drug delivery approach for endothelial and tumor cells. Nano Lett..

[32] Dicheva B.M., Seynhaeve A.L., Soulie T., Eggermont A.M., Ten Hagen T.L., Koning G.A. (2016). Pharmacokinetics, Tissue Distribution and Therapeutic Effect of Cationic Thermosensitive Liposomal Doxorubicin Upon Mild Hyperthermia. Pharm. Res..

[33] Amin M., Bagheri M., Mansourian M., Jaafari M.R., Ten Hagen T.L. (2018). Regulation of in vivo behavior of TAT-modified liposome by associated protein corona and avidity to tumor cells. Int. J. Nanomed..

[34] Zalba S., Contreras A.M., Haeri A., Ten Hagen T.L., Navarro I., Koning G., Garrido M.J. (2015). Cetuximab-oxaliplatin-liposomes for epidermal growth factor receptor targeted chemotherapy of colorectal cancer. J. Control. Release.

[35] Amin M., Mansourian M., Burgers P.C., Amin B., Jaafari M.R., ten Hagen T.L.M. (2022). Increased Targeting Area in Tumors by Dual-Ligand Modification of Liposomes with RGD and TAT Peptides. Pharmaceutics.

[36] van Lummel M., van Blitterswijk W.J., Vink S.R., Veldman R.J., van der Valk M.A., Schipper D., Dicheva B.M., Eggermont A.M., ten Hagen T.L., Verheij M. (2011). Enriching lipid nanovesicles with short-chain glucosylceramide improves doxorubicin delivery and efficacy in solid tumors. FASEB J..

[37] Pedrosa L.R., van Hell A., Suss R., van Blitterswijk W.J., Seynhaeve A.L., van Cappellen W.A., Eggermont A.M., ten Hagen T.L., Verheij M., Koning G.A. (2013). Improving intracellular doxorubicin delivery through nanoliposomes equipped with selective tumor cell membrane permeabilizing short-chain sphingolipids. Pharm. Res..

[38] Cordeiro Pedrosa L.R., van Cappellen W.A., Steurer B., Ciceri D., ten Hagen T.L., Eggermont A.M., Verheij M., Goni F.M., Koning G.A., Contreras F.X. (2015). C8-glycosphingolipids preferentially insert into tumor cell membranes and promote chemotherapeutic drug uptake. Biochim. Biophys. Acta.

[39] Zalba S., Seynhaeve A.L.B., Brouwers J.F., Suss R., Verheij M., Ten Hagen T.L.M. (2020). Sensitization of drug resistant sarcoma tumors by membrane modulation via short chain sphingolipid-containing nanoparticles. Nanoscale.

[40] Cordeiro Pedrosa L.R., van Tellingen O., Soullie T., Seynhaeve A.L., Eggermont A.M., Ten Hagen T.L., Verheij M., Koning G.A. (2015). Plasma membrane targeting by short chain sphingolipids inserted in liposomes improves anti-tumor activity of mitoxantrone in an orthotopic breast carcinoma xenograft model. Eur. J. Pharm. Biopharm.

[41] Saeed M., van Brakel M., Zalba S., Schooten E., Rens J.A., Koning G.A., Debets R., Ten Hagen T.L. (2016). Targeting melanoma with immunoliposomes coupled to anti-MAGE A1 TCR-like single-chain antibody. Int. J. Nanomed..

[42] Saeed M., Zalba S., Seynhaeve A.L.B., Debets R., Ten Hagen T.L.M. (2019). Liposomes targeted to MHC-restricted antigen improve drug delivery and antimelanoma response. Int. J. Nanomed..

[43] Merino M., Lozano T., Casares N., Lana H., Troconiz I.F., Ten Hagen T.L.M., Kochan G., Berraondo P., Zalba S., Garrido M.J. (2021). Dual activity of PD-L1 targeted Doxorubicin immunoliposomes promoted an enhanced efficacy of the antitumor immune response in melanoma murine model. J. Nanobiotechnol..

[44] Seynhaeve A.L.B., Dicheva B.M., Hoving S., Koning G.A., Ten Hagen T.L.M. (2013). Intact Doxil is taken up intracellularly and released doxorubicin sequesters in the lysosome: Evaluated by in vitro/in vivo live cell imaging. J. Control. Release.

[45] Seynhaeve A.L.B., Ten Hagen T.L.M. (2021). An adapted dorsal skinfold model used for 4D intravital followed by whole-mount imaging to reveal endothelial cell-pericyte association. Sci. Rep..

[46] Seynhaeve A.L.B., Ten Hagen T.L.M. (2018). Intravital Microscopy of Tumor-associated Vasculature Using Advanced Dorsal Skinfold Window Chambers on Transgenic Fluorescent Mice. J. Vis. Exp..

[47] Seynhaeve A.L.B., Oostinga D., van Haperen R., Eilken H.M., Adams S., Adams R.H., Ten Hagen T.L.M. (2018). Spatiotemporal endothelial cell—Pericyte association in tumors as shown by high resolution 4D intravital imaging. Sci. Rep..

[48] Dreher M.R., Liu W., Michelich C.R., Dewhirst M.W., Yuan F., Chilkoti A. (2006). Tumor vascular permeability, accumulation, and penetration of macromolecular drug carriers. J. Natl. Cancer Inst..

[49] Manzoor A.A., Lindner L.H., Landon C.D., Park J.Y., Simnick A.J., Dreher M.R., Das S., Hanna G., Park W., Chilkoti A. (2012). Overcoming limitations in nanoparticle drug delivery: Triggered, intravascular release to improve drug penetration into tumors. Cancer Res..

[50] Seynhaeve A.L., Hoving S., Schipper D., Vermeulen C.E., de Wiel-Ambagtsheer G., van Tiel S.T., Eggermont A.M., Ten Hagen T.L. (2007). Tumor necrosis factor alpha mediates homogeneous distribution of liposomes in murine melanoma that contributes to a better tumor response. Cancer Res..

[51] Lu T., Lokerse W.J.M., Seynhaeve A.L.B., Koning G.A., Ten Hagen T.L.M. (2015). Formulation and optimization of idarubicin thermosensitive liposomes provides ultrafast triggered release at mild hyperthermia and improves tumor response. J. Control. Release.

[52] Pedrosa L.R., Ten Hagen T.L., Suss R., van Hell A., Eggermont A.M., Verheij M., Koning G.A. (2015). Short-chain glycoceramides promote intracellular mitoxantrone delivery from novel nanoliposomes into breast cancer cells. Pharm. Res..

[53] Djanashvili K., ten Hagen T.L., Blange R., Schipper D., Peters J.A., Koning G.A. (2011). Development of a liposomal delivery system for temperature-triggered release of a tumor targeting agent, Ln(III)-DOTA-phenylboronate. Bioorg. Med. Chem..

[54] Dicheva B.M., ten Hagen T.L., Schipper D., Seynhaeve A.L., van Rhoon G.C., Eggermont A.M., Koning G.A. (2014). Targeted and heat-triggered doxorubicin delivery to tumors by dual targeted cationic thermosensitive liposomes. J. Control. Release.

[55] Hobbs S.K., Monsky W.L., Yuan F., Roberts W.G., Griffith L., Torchilin V.P., Jain R.K. (1998). Regulation of transport pathways in tumor vessels: Role of tumor type and microenvironment. Proc. Natl. Acad. Sci. USA.

[56] Hashizume H., Baluk P., Morikawa S., McLean J.W., Thurston G., Roberge S., Jain R.K., McDonald D.M. (2000). Openings between defective endothelial cells explain tumor vessel leakiness. Am. J. Pathol..

[57] Schiffelers R.M., Koning G.A., ten Hagen T.L., Fens M.H., Schraa A.J., Janssen A.P., Kok R.J., Molema G., Storm G. (2003). Anti-tumor efficacy of tumor vasculature-targeted liposomal doxorubicin. J. Control. Release.

[58] Priester M.I., Curto S., Seynhaeve A.L.B., Perdomo A.C., Amin M., Agnass P., Salimibani M., Faridi P., Prakash P., van Rhoon G.C. (2021). Preclinical Studies in Small Animals for Advanced Drug Delivery Using Hyperthermia and Intravital Microscopy. Cancers.

[59] Seynhaeve A.L.B., Amin M., Haemmerich D., van Rhoon G.C., Ten Hagen T.L.M. (2020). Hyperthermia and smart drug delivery systems for solid tumor therapy. Adv. Drug Deliv. Rev..

[60] Chauhan V.P., Stylianopoulos T., Boucher Y., Jain R.K. (2011). Delivery of molecular and nanoscale medicine to tumors: Transport barriers and strategies. Annu. Rev. Chem. Biomol. Eng..

[61] Grunhagen D.J., de Wilt J.H., ten Hagen T.L., Eggermont A.M. (2006). Technology insight: Utility of TNF-alpha-based isolated limb perfusion to avoid amputation of irresectable tumors of the extremities. Nat. Clin. Pract. Oncol..

[62] Lejeune F., Lienard D., Eggermont A. (1998). Regional administration of recombinant tumour necrosis factor-alpha in cancer, with special reference to melanoma. BioDrugs.

[63] Cherix S., Speiser M., Matter M., Raffoul W., Lienard D., Theumann N., Mouhsine E., Mirimanoff R.O., Leyvraz S., Lejeune F.J. (2008). Isolated limb perfusion with tumor necrosis factor and melphalan for non-resectable soft tissue sarcomas: Long-term results on efficacy and limb salvage in a selected group of patients. J. Surg. Oncol..

[64] Brunstein F., Santos I.D., Ferreira L.M., van Tiel S.T., Eggermont A.M., Ten Hagen T.L. (2005). Histamine combined with melphalan in isolated limb perfusion for the treatment of locally advanced soft tissue sarcomas: Preclinical studies in rats. Acta Cir. Bras..

[65] Hoving S., Brunstein F., aan de Wiel-Ambagtsheer G., van Tiel S.T., de Boeck G., de Bruijn E.A., Eggermont A.M., ten Hagen T.L. (2005). Synergistic antitumor response of interleukin 2 with melphalan in isolated limb perfusion in soft tissue sarcoma-bearing rats. Cancer Res..

[66] Hoving S., Seynhaeve A.L., van Tiel S.T., aan de Wiel-Ambagtsheer G., de Bruijn E.A., Eggermont A.M., ten Hagen T.L. (2006). Early destruction of tumor vasculature in tumor necrosis factor-alpha-based isolated limb perfusion is responsible for tumor response. Anticancer Drugs.

[67] van der Veen A.H., de Wilt J.H., Eggermont A.M., van Tiel S.T., Seynhaeve A.L., ten Hagen T.L. (2000). TNF-alpha augments intratumoural concentrations of doxorubicin in TNF-alpha-based isolated limb perfusion in rat sarcoma models and enhances anti-tumour effects. Br. J. Cancer.

[68] de Wilt J.H., ten Hagen T.L., de Boeck G., van Tiel S.T., de Bruijn E.A., Eggermont A.M. (2000). Tumour necrosis factor alpha increases melphalan concentration in tumour tissue after isolated limb perfusion. Br. J. Cancer.

[69] Verhoef C., de Wilt J.H., Grunhagen D.J., van Geel A.N., ten Hagen T.L., Eggermont A.M. (2007). Isolated limb perfusion with melphalan and TNF-alpha in the treatment of extremity sarcoma. Curr. Treat. Options Oncol..

[70] van Rhoon G.C., Franckena M., ten Hagen T.L.M. (2020). A moderate thermal dose is sufficient for effective free and TSL based thermochemotherapy. Adv. Drug Deliv. Rev..

[71] Kong L., Chen Q., Campbell F., Snaar-Jagalska E., Kros A. (2020). Light-Triggered Cancer Cell Specific Targeting and Liposomal Drug Delivery in a Zebrafish Xenograft Model. Adv. Healthc. Mater..

[72] Hoving S., Seynhaeve A.L., van Tiel S.T., Eggermont A.M., ten Hagen T.L. (2005). Addition of low-dose tumor necrosis factor-alpha to systemic treatment with STEALTH liposomal doxorubicin (Doxil) improved anti-tumor activity in osteosarcoma-bearing rats. Anticancer Drugs.

[73] Ten Hagen T.L., Van Der Veen A.H., Nooijen P.T., Van Tiel S.T., Seynhaeve A.L., Eggermont A.M. (2000). Low-dose tumor necrosis factor-alpha augments antitumor activity of stealth liposomal doxorubicin (DOXIL) in soft tissue sarcoma-bearing rats. Int. J. Cancer.

[74] Brouckaert P., Takahashi N., van Tiel S.T., Hostens J., Eggermont A.M.M., Seynhaeve A.L.B., Fiers W., ten Hagen T.L.M. (2004). Tumor necrosis factor-α augmented tumor response in B16BL6 melanoma-bearing mice treated with stealth liposomal doxorubicin (Doxil^®^) correlates with altered Doxil^®^ pharmacokinetics. Int. J. Cancer.

[75] Seetharamu N., Kim E., Hochster H., Martin F., Muggia F. (2010). Phase II study of liposomal cisplatin (SPI-77) in platinum-sensitive recurrences of ovarian cancer. Anticancer Res..

[76] Lee E.S., Gao Z., Bae Y.H. (2008). Recent progress in tumor pH targeting nanotechnology. J. Control. Release.

[77] Yatvin M.B., Kreutz W., Horwitz B.A., Shinitzky M. (1980). pH-sensitive liposomes: Possible clinical implications. Science.

[78] Feng L.Z., Dong Z.L., Tao D.L., Zhang Y.C., Liu Z. (2018). The acidic tumor microenvironment: A target for smart cancer nano-theranostics. Natl. Sci. Rev..

[79] Andresen T.L., Thompson D.H., Kaasgaard T. (2010). Enzyme-triggered nanomedicine: Drug release strategies in cancer therapy. Mol. Membr. Biol..

[80] Ong W., Yang Y., Cruciano A.C., McCarley R.L. (2008). Redox-triggered contents release from liposomes. J. Am. Chem. Soc..

[81] Mura S., Nicolas J., Couvreur P. (2013). Stimuli-responsive nanocarriers for drug delivery. Nat. Mater..

[82] Senapati S., Mahanta A.K., Kumar S., Maiti P. (2018). Controlled drug delivery vehicles for cancer treatment and their performance. Signal Transduct. Target. Ther..

[83] Mo S., Coussios C.C., Seymour L., Carlisle R. (2012). Ultrasound-enhanced drug delivery for cancer. Expert Opin. Drug Deliv..

[84] Eggen S., Fagerland S.M., Morch Y., Hansen R., Sovik K., Berg S., Furu H., Bohn A.D., Lilledahl M.B., Angelsen A. (2014). Ultrasound-enhanced drug delivery in prostate cancer xenografts by nanoparticles stabilizing microbubbles. J. Control. Release.

[85] Huang S.L. (2008). Liposomes in ultrasonic drug and gene delivery. Adv. Drug Deliv. Rev..

[86] Leung S.J., Romanowski M. (2012). Light-activated content release from liposomes. Theranostics.

[87] Bansal A., Zhang Y. (2014). Photocontrolled nanoparticle delivery systems for biomedical applications. Acc. Chem. Res..

[88] Li L., Ten Hagen T.L., Bolkestein M., Gasselhuber A., Yatvin J., van Rhoon G.C., Eggermont A.M., Haemmerich D., Koning G.A. (2013). Improved intratumoral nanoparticle extravasation and penetration by mild hyperthermia. J. Control. Release.

[89] Gaber M.H., Wu N.Z., Hong K., Huang S.K., Dewhirst M.W., Papahadjopoulos D. (1996). Thermosensitive liposomes: Extravasation and release of contents in tumor microvascular networks. Int. J. Radiat. Oncol. Biol. Phys..

[90] Nittayacharn P., Yuan H.X., Hernandez C., Bielecki P., Zhou H., Exner A.A. (2019). Enhancing Tumor Drug Distribution With Ultrasound-Triggered Nanobubbles. J. Pharm. Sci..

[91] Rizzitelli S., Giustetto P., Cutrin J.C., Delli Castelli D., Boffa C., Ruzza M., Menchise V., Molinari F., Aime S., Terreno E. (2015). Sonosensitive theranostic liposomes for preclinical in vivo MRI-guided visualization of doxorubicin release stimulated by pulsed low intensity non-focused ultrasound. J. Control. Release.

[92] Griffin R.J., Ogawa A., Williams B.W., Song C.W. (2005). Hyperthermic enhancement of tumor radiosensitization strategies. Immunol. Investig..

[93] Griffin R.J., Dings R.P., Jamshidi-Parsian A., Song C.W. (2010). Mild temperature hyperthermia and radiation therapy: Role of tumour vascular thermotolerance and relevant physiological factors. Int. J. Hyperth..

[94] Lepock J.R. (2003). Cellular effects of hyperthermia: Relevance to the minimum dose for thermal damage. Int. J. Hyperth..

[95] Roti J.L.R. (2008). Cellular responses to hyperthermia (40-46 degrees C): Cell killing and molecular events. Int. J. Hyperth..

[96] Overgaard J., Grau C., Lindegaard J.C., Horsman M.R. (1991). The Potential of Using Hyperthermia to Eliminate Radioresistant Hypoxic Cells. Radiother. Oncol..

[97] Oei A.L., Vriend L.E.M., Crezee J., Franken N.A.P., Krawczyk P.M. (2015). Effects of hyperthermia on DNA repair pathways: One treatment to inhibit them all. Radiat Oncol..

[98] Skitzki J.J., Repasky E.A., Evans S.S. (2009). Hyperthermia as an immunotherapy strategy for cancer. Curr. Opin Investig. Dr..

[99] Lee S., Son B., Park G., Kim H., Kang H., Jeon J., Youn H., Youn B. (2018). Immunogenic Effect of Hyperthermia on Enhancing Radiotherapeutic Efficacy. Int. J. Mol. Sci..

[100] Toraya-Brown S., Sheen M.R., Zhang P., Chen L., Baird J.R., Demidenko E., Turk M.J., Hoopes P.J., Conejo-Garcia J.R., Fiering S. (2014). Local hyperthermia treatment of tumors induces CD8(+) T cell-mediated resistance against distal and secondary tumors. Nanomedicine.

[101] Tsang Y.W., Huang C.C., Yang K.L., Chi M.S., Chiang H.C., Wang Y.S., Andocs G., Szasz A., Li W.T., Chi K.H. (2015). Improving immunological tumor microenvironment using electro-hyperthermia followed by dendritic cell immunotherapy. BMC Cancer.

[102] Issels R.D., Lindner L.H., Verweij J., Wust P., Reichardt P., Schem B.C., Abdel-Rahman S., Daugaard S., Salat C., Wendtner C.M. (2010). Neo-adjuvant chemotherapy alone or with regional hyperthermia for localised high-risk soft-tissue sarcoma: A randomised phase 3 multicentre study. Lancet Oncol..

[103] Issels R.D., Lindner L.H., Verweij J., Wessalowski R., Reichardt P., Wust P., Ghadjar P., Hohenberger P., Angele M., Salat C. (2018). Effect of Neoadjuvant Chemotherapy Plus Regional Hyperthermia on Long-term Outcomes Among Patients With Localized High-Risk Soft Tissue Sarcoma: The EORTC 62961-ESHO 95 Randomized Clinical Trial. JAMA Oncol..

[104] Killock D. (2018). Sarcoma: Local hyperthermia improves survival. Nat. Rev. Clin. Oncol..

[105] Issels R., Kampmann E., Kanaar R., Lindner L.H. (2016). Hallmarks of hyperthermia in driving the future of clinical hyperthermia as targeted therapy: Translation into clinical application. Int. J. Hyperth..

[106] Huang S.K., Stauffer P.R., Hong K., Guo J.W., Phillips T.L., Huang A., Papahadjopoulos D. (1994). Liposomes and hyperthermia in mice: Increased tumor uptake and therapeutic efficacy of doxorubicin in sterically stabilized liposomes. Cancer Res..

[107] Kong G., Braun R.D., Dewhirst M.W. (2000). Hyperthermia enables tumor-specific nanoparticle delivery: Effect of particle size. Cancer Res..

[108] Kong G., Braun R.D., Dewhirst M.W. (2001). Characterization of the effect of hyperthermia on nanoparticle extravasation from tumor vasculature. Cancer Res..

[109] Amin M., Huang W., Seynhaeve A.L.B., Ten Hagen T.L.M. (2020). Hyperthermia and Temperature-Sensitive Nanomaterials for Spatiotemporal Drug Delivery to Solid Tumors. Pharmaceutics.

[110] Amin M., Lammers T., Ten Hagen T.L.M. (2022). Temperature-sensitive polymers to promote heat-triggered drug release from liposomes: Towards bypassing EPR. Adv. Drug Deliv. Rev..

[111] Papahadjopoulos D., Jacobson K., Nir S., Isac T. (1973). Phase transitions in phospholipid vesicles. Fluorescence polarization and permeability measurements concerning the effect of temperature and cholesterol. Biochim. Biophys. Acta.

[112] Phillips M.C., Hauser H., Paltauf F. (1972). The inter- and intra-molecular mixing of hydrocarbon chains in lecithin-water systems. Chem. Phys. Lipids.

[113] Ten Hagen T.L.M., Dreher M.R., Zalba S., Seynhaeve A.L.B., Amin M., Li L., Haemmerich D. (2021). Drug transport kinetics of intravascular triggered drug delivery systems. Commun. Biol..

[114] Yatvin M.B., Weinstein J.N., Dennis W.H., Blumenthal R. (1978). Design of liposomes for enhanced local release of drugs by hyperthermia. Science.

[115] Weinstein J.N., Magin R.L., Yatvin M.B., Zaharko D.S. (1979). Liposomes and local hyperthermia: Selective delivery of methotrexate to heated tumors. Science.

[116] Yatvin M.B., Muhlensiepen H., Porschen W., Weinstein J.N., Feinendegen L.E. (1981). Selective delivery of liposome-associated cis-dichlorodiammineplatinum(II) by heat and its influence on tumor drug uptake and growth. Cancer Res..

[117] Iga K., Hamaguchi N., Igari Y., Ogawa Y., Gotoh K., Ootsu K., Toguchi H., Shimamoto T. (1991). Enhanced antitumor activity in mice after administration of thermosensitive liposome encapsulating cisplatin with hyperthermia. J. Pharmacol. Exp. Ther..

[118] Maruyama K., Unezaki S., Takahashi N., Iwatsuru M. (1993). Enhanced delivery of doxorubicin to tumor by long-circulating thermosensitive liposomes and local hyperthermia. Biochim. Biophys. Acta.

[119] Gaber M.H., Hong K., Huang S.K., Papahadjopoulos D. (1995). Thermosensitive sterically stabilized liposomes: Formulation and in vitro studies on mechanism of doxorubicin release by bovine serum and human plasma. Pharm. Res..

[120] Sadeghi N., Deckers R., Ozbakir B., Akthar S., Kok R.J., Lammers T., Storm G. (2018). Influence of cholesterol inclusion on the doxorubicin release characteristics of lysolipid-based thermosensitive liposomes. Int. J. Pharm..

[121] Landon C.D., Park J.Y., Needham D., Dewhirst M.W. (2011). Nanoscale Drug Delivery and Hyperthermia: The Materials Design and Preclinical and Clinical Testing of Low Temperature-Sensitive Liposomes Used in Combination with Mild Hyperthermia in the Treatment of Local Cancer. Open Nanomed. J..

[122] Li L., ten Hagen T.L., Hossann M., Suss R., van Rhoon G.C., Eggermont A.M., Haemmerich D., Koning G.A. (2013). Mild hyperthermia triggered doxorubicin release from optimized stealth thermosensitive liposomes improves intratumoral drug delivery and efficacy. J. Control. Release.

[123] Anyarambhatla G.R., Needham D. (1999). Enhancement of the phase transition permeability of DPPC liposomes by incorporation of MPPC: A new temperature-sensitive liposome for use with mild hyperthermia. J. Liposome Res..

[124] Mills J.K., Needham D. (2005). Lysolipid incorporation in dipalmitoylphosphatidylcholine bilayer membranes enhances the ion permeability and drug release rates at the membrane phase transition. Biochim. Biophys. Acta.

[125] Lindner L.H., Eichhorn M.E., Eibl H., Teichert N., Schmitt-Sody M., Issels R.D., Dellian M. (2004). Novel temperature-sensitive liposomes with prolonged circulation time. Clin. Cancer Res..

[126] Hossann M., Wiggenhorn M., Schwerdt A., Wachholz K., Teichert N., Eibl H., Issels R.D., Lindner L.H. (2007). In vitro stability and content release properties of phosphatidylglyceroglycerol containing thermosensitive liposomes. Biochim. Biophys. Acta.

[127] Brummelhuis I.S.G., Simons M., Lindner L.H., Kort S., de Jong S., Hossann M., Witjes J.A., Oosterwijk E. (2021). DPPG2-based thermosensitive liposomes as drug delivery system for effective muscle-invasive bladder cancer treatment in vivo. Int. J. Hyperth..

[128] Gasselhuber A., Dreher M.R., Rattay F., Wood B.J., Haemmerich D. (2012). Comparison of conventional chemotherapy, stealth liposomes and temperature-sensitive liposomes in a mathematical model. PLoS ONE.

[129] Chen Q., Krol A., Wright A., Needham D., Dewhirst M.W., Yuan F. (2008). Tumor microvascular permeability is a key determinant for antivascular effects of doxorubicin encapsulated in a temperature sensitive liposome. Int. J. Hyperth..

[130] Chen Q., Tong S., Dewhirst M.W., Yuan F. (2004). Targeting tumor microvessels using doxorubicin encapsulated in a novel thermosensitive liposome. Mol. Cancer Ther..

[131] Al-Jamal W.T., Kostarelos K. (2022). Mild hyperthermia accelerates doxorubicin clearance from tumour-extravasated temperature-sensitive liposomes. Nanotheranostics.

[132] Poon R.T., Borys N. (2009). Lyso-thermosensitive liposomal doxorubicin: A novel approach to enhance efficacy of thermal ablation of liver cancer. Expert Opin. Pharmacother..

[133] Dou Y., Hynynen K., Allen C. (2017). To heat or not to heat: Challenges with clinical translation of thermosensitive liposomes. J. Control. Release.

[134] Lokerse W.J.M., Lazarian A., Kleinhempel A., Petrini M., Schwarz P., Hossann M., Holdt L.M., Mailander V., Lindner L.H. (2021). Mechanistic investigation of thermosensitive liposome immunogenicity and understanding the drivers for circulation half-life: A polyethylene glycol versus 1,2-dipalmitoyl-sn-glycero-3-phosphodiglycerol study. J. Control. Release.

[135] van Valenberg F.J.P., Brummelhuis I.S.G., Lindner L.H., Kuhnle F., Wedmann B., Schweizer P., Hossann M., Witjes J.A., Oosterwijk E. (2021). DPPG2-Based Thermosensitive Liposomes with Encapsulated Doxorubicin Combined with Hyperthermia Lead to Higher Doxorubicin Concentrations in the Bladder Compared to Conventional Application in Pigs: A Rationale for the Treatment of Muscle-Invasive Bladder Cancer. Int. J. Nanomed..

[136] Harrington K.J., Mohammadtaghi S., Uster P.S., Glass D., Peters A.M., Vile R.G., Stewart J.S. (2001). Effective targeting of solid tumors in patients with locally advanced cancers by radiolabeled pegylated liposomes. Clin. Cancer Res..

[137] Uziely B., Jeffers S., Isacson R., Kutsch K., Wei-Tsao D., Yehoshua Z., Libson E., Muggia F.M., Gabizon A. (1995). Liposomal doxorubicin: Antitumor activity and unique toxicities during two complementary phase I studies. J. Clin. Oncol..

[138] Gabizon A.A., Patil Y., La-Beck N.M. (2016). New insights and evolving role of pegylated liposomal doxorubicin in cancer therapy. Drug Resist. Updat..

[139] Anchordoquy T.J., Barenholz Y., Boraschi D., Chorny M., Decuzzi P., Dobrovolskaia M.A., Farhangrazi Z.S., Farrell D., Gabizon A., Ghandehari H. (2017). Mechanisms and Barriers in Cancer Nanomedicine: Addressing Challenges, Looking for Solutions. ACS Nano.

[140] Denekamp J. (1984). Vascular endothelium as the vulnerable element in tumours. Acta Radiol. Oncol..

[141] Corti A., Pastorino F., Curnis F., Arap W., Ponzoni M., Pasqualini R. (2012). Targeted Drug Delivery and Penetration Into Solid Tumors. Med. Res. Rev..

[142] Maeda N., Takeuchi Y., Takada M., Sadzuka Y., Namba Y., Oku N. (2004). Anti-neovascular therapy by use of tumor neovasculature-targeted long-circulating liposome. J. Control. Release.

[143] Oku N., Asai T., Watanabe K., Kuromi K., Nagatsuka M., Kurohane K., Kikkawa H., Ogino K., Tanaka M., Ishikawa D. (2002). Anti-neovascular therapy using novel peptides homing to angiogenic vessels. Oncogene.

[144] Alessi P., Ebbinghaus C., Neri D. (2004). Molecular targeting of angiogenesis. Biochim. Biophys. Acta-Rev. Cancer.

[145] Bikfalvi A., Bicknell R. (2002). Recent advances in angiogenesis, anti-angiogenesis and vascular targeting. Trends Pharmacol. Sci..

[146] Eichhorn M.E., Strieth S., Dellian M. (2004). Anti-vascular tumor therapy: Recent advances, pitfalls and clinical perspectives. Drug Resist. Updates.

[147] Ruoslahti E. (2003). The RGD story: A personal account. Matrix Biol..

[148] Heckmann D., Meyer A., Marinelli L., Zahn G., Stragies R., Kessler H. (2007). Probing integrin selectivity: Rational design of highly active and selective ligands for the alpha 5 beta 1 and alpha v beta 3 integrin receptor. Angew. Chem. Int. Ed..

[149] Temming K., Schiffelers R.M., Molema G., Kok R.J. (2005). RGD-based strategies for selective delivery of therapeutics and imaging agents to the tumour vasculature. Drug Resist. Updat.

[150] Salvati M., Cordero F.M., Pisaneschi F., Melani F., Gratteri P., Cini N., Bottoncetti A., Brandi A. (2008). Synthesis, SAR and in vitro evaluation of new cyclic Arg-Gly-Asp pseudopentapeptides containing a s-cis peptide bond as integrin alphavbeta3 and alphavbeta5 ligands. Bioorg. Med. Chem..

[151] Heckmann D., Kesster H. (2007). Design and chemical synthesis of integrin ligands. Integrins.

[152] Amin M., Badiee A., Jaafari M.R. (2013). Improvement of pharmacokinetic and antitumor activity of PEGylated liposomal doxorubicin by targeting with N-methylated cyclic RGD peptide in mice bearing C-26 colon carcinomas. Int. J. Pharm..

[153] Vincent S., DePace D., Finkelstein S. (1988). Distribution of anionic sites on the capillary endothelium in an experimental brain tumor model. Microcirc Endothel. Lymphat..

[154] Ran S., Downes A., Thorpe P.E. (2002). Increased exposure of anionic phospholipids on the surface of tumor blood vessels. Cancer Res..

[155] Ho E.A., Ramsay E., Ginj M., Anantha M., Bregman I., Sy J., Woo J., Osooly-Talesh M., Yapp D.T., Bally M.B. (2010). Characterization of cationic liposome formulations designed to exhibit extended plasma residence times and tumor vasculature targeting properties. J. Pharm. Sci..

[156] Torchilin V.P., Rammohan R., Weissig V., Levchenko T.S. (2001). TAT peptide on the surface of liposomes affords their efficient intracellular delivery even at low temperature and in the presence of metabolic inhibitors. Proc. Natl. Acad. Sci. USA.

[157] Fretz M.M., Koning G.A., Mastrobattista E., Jiskoot W., Storm G. (2004). OVCAR-3 cells internalize TAT-peptide modified liposomes by endocytosis. Biochim. Biophys. Acta.

[158] Seguin J., Nicolazzi C., Mignet N., Scherman D., Chabot G.G. (2012). Vascular density and endothelial cell expression of integrin alpha v beta 3 and E-selectin in murine tumours. Tumour Biol..

[159] Contreras F.X., Villar A.V., Alonso A., Kolesnick R.N., Goni F.M. (2003). Sphingomyelinase activity causes transbilayer lipid translocation in model and cell membranes. J. Biol. Chem..

[160] Contreras F.X., Basanez G., Alonso A., Herrmann A., Goni F.M. (2005). Asymmetric addition of ceramides but not dihydroceramides promotes transbilayer (flip-flop) lipid motion in membranes. Biophys. J..

[161] Veldman R.J., Zerp S., van Blitterswijk W.J., Verheij M. (2004). N-hexanoyl-sphingomyelin potentiates in vitro doxorubicin cytotoxicity by enhancing its cellular influx. Br. J. Cancer.

[162] Khalil D.N., Smith E.L., Brentjens R.J., Wolchok J.D. (2016). The future of cancer treatment: Immunomodulation, CARs and combination immunotherapy. Nat. Rev. Clin. Oncol..

[163] Song C.W., Kim H., Cho H., Kim M.S., Paek S.H., Park H.J., Griffin R.J., Terezakis S., Cho L.C. (2022). HIF-1 alpha Inhibition Improves Anti-Tumor Immunity and Promotes the Efficacy of Stereotactic Ablative Radiotherapy (SABR). Cancers.

[164] Rastakhiz S., Yazdani M., Shariat S., Arab A., Momtazi-Borojeni A.A., Barati N., Mansourian M., Amin M., Abbasi A., Saberi Z. (2019). Preparation of nanoliposomes linked to HER2/neu-derived (P5) peptide containing MPL adjuvant as vaccine against breast cancer. J. Cell Biochem..

[165] Ding Y., Wang L., Li H., Miao F., Zhang Z., Hu C., Yu W., Tang Q., Shao G. (2022). Application of lipid nanovesicle drug delivery system in cancer immunotherapy. J. Nanobiotechnol..

[166] Gu Z., Da Silva C.G., Van der Maaden K., Ossendorp F., Cruz L.J. (2020). Liposome-Based Drug Delivery Systems in Cancer Immunotherapy. Pharmaceutics.

[167] Fobian S.F., Cheng Z., Ten Hagen T.L.M. (2021). Smart Lipid-Based Nanosystems for Therapeutic Immune Induction against Cancers: Perspectives and Outlooks. Pharmaceutics.

[168] Saeed M., Schooten E., van Brakel M., Cole D.K., Ten Hagen T.L.M., Debets R. (2020). T Cells Expressing a TCR-Like Antibody Selected Against the Heteroclitic Variant of a Shared MAGE-A Epitope Do Not Recognise the Cognate Epitope. Cancers.

[169] Merino M., Contreras A., Casares N., Troconiz I.F., Ten Hagen T.L., Berraondo P., Zalba S., Garrido M.J. (2019). A new immune-nanoplatform for promoting adaptive antitumor immune response. Nanomedicine.

